# The effects of magnesium L-threonate (Magtein^®^) on cognitive performance and sleep quality in adults: a randomised, double-blind, placebo-controlled trial

**DOI:** 10.3389/fnut.2025.1729164

**Published:** 2026-01-12

**Authors:** Adrian L. Lopresti, Stephen J. Smith

**Affiliations:** 1 Clinical Research Australia, Perth, WA, Australia; 2 College of Science, Health, Engineering and Education, Murdoch University, Perth, WA, Australia

**Keywords:** brain aging, clinical trial, cognition, magnesium L-threonate, Magtein^®^, memory, NIH toolbox, sleep

## Abstract

**Background/objectives:**

Magnesium may help support cognition and sleep. The purpose of this two-arm, 6-week, parallel-group, randomised, double-blind, placebo-controlled trial was to examine the effects of magnesium L-threonate (Magtein^®^) supplementation on cognitive performance, cognitive age, sleep quality, and selected physiological indicators in adults.

**Methods:**

One hundred adults aged 18 to 45 with self-reported dissatisfied sleep were supplemented with 2 g daily of Magtein^®^ or a placebo. Outcome measures comprised the computer-based National Institute for Health (NIH) Cognitive Toolbox and Raven’s Progressive Matrices Version 2 for the assessment of cognitive function, self-report evaluations of sleep quality and emotional wellbeing, a reaction time test, and physiological data obtained from a sleep-tracking wearable device (Oura Ring), including resting heart rate (HR) and heart rate variability (HRV) during sleep.

**Results:**

Compared to the placebo, Magtein^®^ was associated with greater improvements in overall cognitive performance as measured by the NIH Total Cognition Composite (*p* = 0.043), with larger treatment effects on working and episodic memory. There was also a 7.5-year reduction in estimated brain cognitive age and a greater improvement in reaction time (*p* = 0.031). However, there were no group differences in changes in the Raven’s test (*p* = 0.953). Based on self-report measures, there was a greater improvement in sleep-related impairment (*p* = 0.043), but no group differences in changes in sleep disturbances (*p* = 0.316), restorative sleep (*p* = 0.439), or general wellbeing (*p* = 0.436); although in a subset of participants with more severe sleep-related problems, group differences in sleep-disturbances were identified (*p* = 0.031). Based on data from the sleep tracking ring, there were no group differences in sleep outcomes, although there was a greater reduction in HR (*p* = 0.030) and an increase in HRV (*p* = 0.036), a physiological marker of stress reduction and improved autonomic balance. Magtein^®^ was well-tolerated, and there were no reports of significant adverse reactions.

**Conclusion:**

The results from this study suggest Magtein^®^ supplementation for 6 weeks improves overall cognition, cognitive age, working memory, reaction time, HR, HRV, and some subjective, but not objective measures of sleep in healthy adults with self-reported dissatisfied sleep.

## 1 Introduction

Magnesium is a mineral that has numerous essential roles in the body. It is required as a cofactor in more than 300 enzymatic reactions that are important for energy generation, cardiovascular health, neuromuscular function, bone and teeth maintenance, cognition, and nervous system function (1, 2). Foods rich in magnesium include grains, cereals, nuts, and dark leafy vegetables. Unfortunately, research confirms that approximately 50% of the US population does not consume the recommended daily dietary requirement of magnesium (3), and roughly 30% of the global population has an inadequate dietary magnesium intake (4). Additionally, the decline in magnesium concentrations in modern food crops may contribute to reduced dietary magnesium intake, potentially increasing the risk of magnesium deficiency and chronic health conditions, such as cardiovascular disease and metabolic syndrome (5).

Low blood magnesium concentrations have been identified in many conditions, such as depression, anxiety, cardiovascular diseases, cardio-metabolic syndromes, and type 2 diabetes (6). Moreover, lower blood concentrations of magnesium have been identified in adults with reduced cognitive performance, mild cognitive impairment, and Alzheimer’s disease (7–9). However, it is important to note that these correlational studies do not confirm causality, as these health conditions are associated with several factors that may influence magnesium status, such as poorer dietary habits or gastrointestinal changes that affect magnesium absorption (10, 11). Based on results from the National Health and Nutrition Survey, which comprised 2,508 participants aged 60 years and older, a higher magnesium intake was independently associated with higher global cognitive scores (12). However, despite research demonstrating an association between low magnesium concentrations in the body and poorer cognitive performance, trials investigating whether magnesium repletion through dietary interventions or supplementation can enhance cognitive performance are limited. This may be partly attributed to the fact that most magnesium supplements have limited blood–brain permeability, resulting in minimal increases in brain concentrations of magnesium despite elevated blood concentrations (13–16). This may result in reduced cognitive-related benefits.

In a randomised, double-blind, placebo-controlled study on healthy Chinese adults aged 18 to 65, 30 days of supplementation with Magtein^®^, phosphatidylserine, and vitamins D3, C, and B6 was associated with memory improvements, which were more pronounced in older participants (17). In an open-label study on adults with attention-deficit hyperactivity disorder (ADHD), 12 weeks of Magtein^®^ supplementation was associated with improvements in self-reported and clinician-rated measures of attention, and objective measures of cognitive performance and intelligence (18). In another 12-week randomised controlled trial on older adults with mild cognitive impairment, Magtein^®^ supplementation was associated with a significant improvement in the total cognitive score, which was equivalent to an approximate 9-year cognitive improvement, suggesting a meaningful reversal of age-related cognitive decline (19).

Since research indicates that low blood magnesium concentrations are associated with reduced cognitive performance, and there is preliminary evidence that supplementation may improve cognitive skills, the primary objective of this study was to examine the effects of 6 weeks of magnesium supplementation (in the form of Magtein^®^) on cognition in healthy young-to-middle-aged adults. To minimise the risk of ceiling effects from magnesium supplementation in cognitively intact, healthy young-to-middle-aged adults, a cohort of participants experiencing self-reported dissatisfied sleep was recruited. This cohort was selected as poor sleep is associated with worsened cognitive performance (20), sleep disturbances can deplete magnesium concentrations, and hypomagnesemia is associated with excessive daytime sleepiness (21–23).

Changes in sleep quality over time were also evaluated over time in the present study. Research confirms an association between poor sleep and magnesium status. In a recent systematic review, it was concluded that based on the findings from 4 observational studies (one cohort and three cross-sectional), low magnesium status was associated with reduced sleep quality (delayed sleep onset, daytime sleepiness, and reduced sleep duration) (24). However, the evidence from randomised controlled trials examining the effects of magnesium supplementation on sleep quality is limited. In this systematic review, only 5 randomised controlled interventional studies were identified (24). Two studies were rated as low quality, two were fair quality, and one was high quality. Sample sizes were small, study designs were often flawed, and three studies were conducted on healthy participants with no reported sleep problems. Moreover, in two of the five studies, magnesium supplementation did not improve sleep quality more than the placebo. Therefore, robust randomised controlled trials examining the effects of magnesium supplementation on sleep quality are required. This is particularly important as magnesium is regularly used by consumers and practitioners to support sleep health.

Finally, in the present study, participants were supplemented with a form of magnesium known as Magtein^®^. Magtein^®^ was the magnesium form used in the previously mentioned studies on cognition (17, 18). Furthermore, animal studies demonstrate that it has greater bioavailability compared to other magnesium supplements and can increase brain magnesium concentrations (14, 15, 25). The L-threonate ligand in Magtein^®^ plays an important functional and mechanistic role in this compound, as preclinical research has demonstrated that the effect of threonate is mediated through glucose transporters, which enhances magnesium bioavailability and enables significant increases in brain magnesium concentrations (15). It was hypothesised that Magtein^®^ supplementation may improve general cognitive performance and sleep quality in adults presenting with dissatisfied sleep.

## 2 Materials and methods

### 2.1 Study design and procedures

Ethics approval was acquired from the National Institute of Integrative Medicine Human Research Ethics Committee, and informed consent was obtained participants before study commencement. This study was registered prospectively with the Australian and New Zealand Clinical Trials Registry (ACTRN12624000379516).

**Figure 1:**
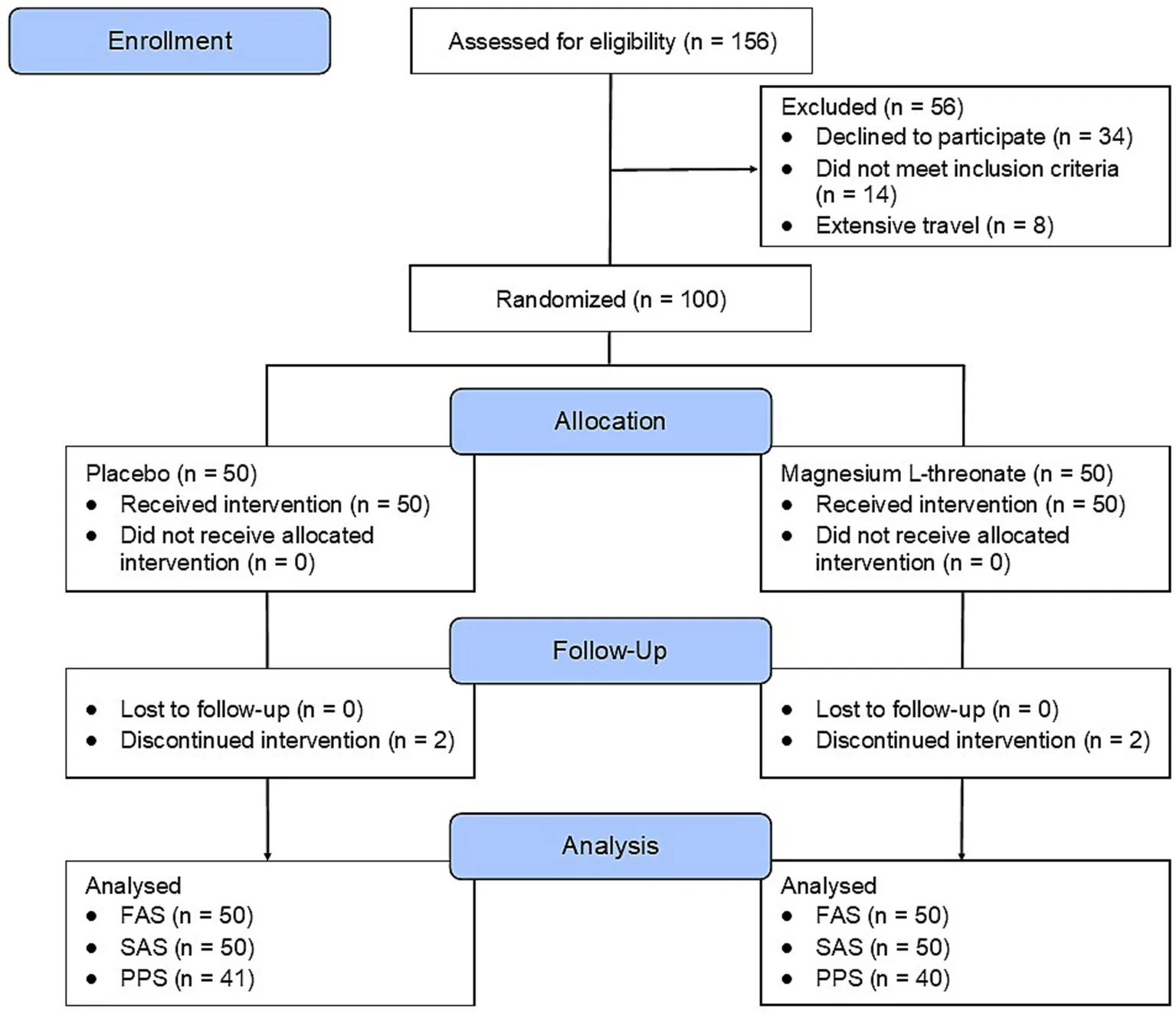
Systematic illustration of study design.

This was a 6-week, two-arm, parallel-group, randomised, double-blind, placebo-controlled trial (Figure 1). Participants attended visit 1 (day −7), where they completed the tests of cognitive performance and all self-report questionnaires. They were also given an Oura Ring (version 3) to wear during sleep and were provided with a supply of their study capsules. Participants were instructed to wear their Oura Ring for 7 days to obtain baseline sleep data. After 7 days (day 1), participants were instructed to commence their study capsules. Self-report questionnaires were completed online on days 14 and 28. Participants returned for a second in-person assessment (visit 2, day 42), after 6 weeks of capsule intake. During this visit, cognitive assessments were re-administered, self-report questionnaires were completed, and study capsules and the Oura Ring were returned. All in-person assessments were conducted in the morning between 8 and 11 a.m., and participants were instructed to not consume more than 2 standard serves of alcohol the evening before assessment visits, to avoid consuming any caffeinated beverage (tea or coffee) on the morning of the assessment, and to not engage in strenuous exercise on the morning of the assessment. The consumption of a light breakfast was permitted, but participants were requested to consume a similar meal on visits 1 and 2.

### 2.2 Randomisation and blinding

Through social media advertisements and emails to an in-house database, volunteer recruitment occurred from April to November 2024. Eligible participants were randomly allocated to either a Magtein^®^ or placebo condition in a 1:1 ratio using a randomisation calculator with a randomisation structure consisting of 10 blocks, with 10 participants per block. Identification numbers were allocated based on the order of enrolment, with the randomisation sequence produced by an investigator not directly involved in volunteer recruitment. All capsules were provided in identical bottles with bottle codes held by the study sponsor until study completion. Researchers and the statistician remained blinded to group allocation until all outcomes were obtained, and a blind review was undertaken.

### 2.3 Participants

#### 2.3.1 Inclusion criteria

Inclusion criteria for the study comprised the following: healthy male and female adults; aged between 18 and 45 years; self-reported symptoms of poor sleep lasting longer than 4 weeks; typical bedtime was between 9 p.m. and 12 a.m.; body mass index (BMI) between 18 and 35 kg/m^2^; non-smoker; and had no plan to start a new treatment during the study period.

#### 2.3.2 Exclusion criteria

The exclusion criteria comprised the following: a diagnosed sleep disorder; neurological condition such as Parkinson’s disease, Alzheimer’s disease, intracranial haemorrhage, or head or brain injury; a diagnosis of a psychiatric disorder; a recent diagnosis or having an unmanaged medical condition, including but not limited to, diabetes, hypertension, cardiovascular disease, autoimmune disease, endocrine disease, cancer/malignancy, or acute or chronic pain condition; regular medication intake, including but not limited to, anticonvulsants, benzodiazepines, opioids, corticosteroids, or immunosuppressants; a medication or nutritional/herbal supplement change in the prior 3 months or an expectation to change during the study; the current use of supplements that contained more than 25 mg of elemental magnesium; coffee intake greater than 3 cups a day (or equivalent caffeine intake from other caffeinated drinks, e.g., tea, energy drinks, etc.); external or lifestyle factors that may affect sleep patterns (e.g., infant/children regularly wakening, excessive noise, a snoring partner, pain condition, variable work or study schedules, and/or mid-to-late afternoon/evening intake of caffeine); planned major lifestyle change in the next 3 months; alcohol intake greater than 14 standard drinks per week; illicit drug use in the past 12 months; pregnant women, women who were breastfeeding, or women who intended to fall pregnant during the study period; or any significant surgeries over the last year.

### 2.4 Interventions

The intervention consisted of either magnesium L-threonate (Magtein^®^) or a placebo (rice flour). Participants took one capsule in the morning and one in the evening (2 h before bedtime, 1 g Magtein^®^ per capsule) with or without food, with the active intervention delivering 2 g of Magtein^®^ (145 mg of elemental magnesium) daily for 6 weeks. This is the dose that has most commonly been used in cognitive trials supplementing with Magtein^®^ (17, 19). Moreover, to maintain steady magnesium concentrations in the body, Magtein^®^ is typically administered twice daily. The active and placebo capsules were similar in appearance, matched for shape, colour, and size, with both capsules containing similar excipients (rice flour, silicon dioxide and stearic acid). Participants returned their remaining bottles and capsules at visit 2, and a capsule count was completed to assess treatment compliance. To evaluate the efficacy of treatment blinding participants predicted their group allocation (placebo, magnesium, or unsure) at visit 2.

### 2.5 Outcome measures

#### 2.5.1 National Institute of Health (NIH) toolbox cognition battery

The NIH Toolbox Cognition Battery is a validated measure of cognitive abilities that consists of tests of multiple constructs (26). It yields individual test scores and composite scores comprising (1) Total Cognition Composite (a combination of fluid composite and crystallised composite), (2) Fluid Composite (tasks comprising Dimensional Change Card Sort, Flanker Inhibitory Control and Attention, Picture Sequence Memory, List Sorting Working Memory, and Pattern Comparison), and (3) Crystallised Composite (tasks comprising Picture Vocabulary and Oral Reading Recognition). The Total Cognition Composite score was established as the primary outcome measure.

As an exploratory analysis, the NIH Toolbox Total Cognition Composite Change Sensitive Score (CSS) was used to calculate “cognitive age.” A cognitive age reflects how “old” someone’s cognitive function appears compared to a normative sample. The concept of cognitive age emerged from a long history of cognitive aging research, which focused on characterising average age-related declines in cognitive abilities such as processing speed, memory, and executive functions using psychometric tests (27). The goal was to quantify an individual’s cognitive performance relative to their peers, translating scores from cognitive tests into an easily interpretable age-equivalent score (28).

#### 2.5.2 Raven’s progressive matrices second edition (Raven’s 2)

The Raven’s 2 is a validated nonverbal assessment that provides a non-verbal estimate of fluid intelligence (the ability to solve novel reasoning problems). Fluid intelligence is correlated with several important skills, such as comprehension, problem-solving, and learning (29). The Raven’s 2 short form, was administered by computer.

#### 2.5.3 PROMIS sleep disturbance and sleep-related impairment scale (PROMIS sleep)

The PROMIS Sleep is a 16-item self-report questionnaire where questions are rated on a 5-point Likert scale. Component scores are calculated for sleep disturbance and sleep-related impairment (30). The PROMIS Sleep questionnaire correlates highly with the Pittsburgh Sleep Quality Index, but has fewer questions (31). Moreover, the PROMIS sleep has been shown to identify individuals with and without self-reported sleep disorders and between those with treated and untreated sleep disorders (30).

#### 2.5.4 Restorative sleep questionnaire (RSQ)

The RSQ was completed online and is a validated, 9-item self-report questionnaire that evaluates restorative sleep using a 5-point Likert scale assessing feelings of tiredness, mood, and energy over the last week (32).

#### 2.5.5 World Health Organisation-5 wellbeing index (WHO-5)

The WHO-5 was completed online and is a 5-item self-report inventory that measures psychological wellbeing (33). Ratings are based on the last 2 weeks using a 5-point Likert scale.

#### 2.5.6 Oura Ring sleep measures

Oura Ring is a wearable device designed to measure changes in sleep patterns. It provides generally reliable and valid readings of total sleep time, sleep efficiency, sleep latency, and time in deep and REM sleep (34). Average resting heart rate and heart rate variability (HRV), as measured by the root mean square of successive differences between normal heartbeats (RMSSD), during sleep, were also measured. To obtain baseline records before supplementation, data from the Oura Ring were collected for 7 days before capsule intake.

#### 2.5.7 3D aim trainer score

3D Aim Trainer^1^ is a first-person shooting game. In the Tile Frenzy task, participants were required to shoot as many tiles as possible in 30 s. A total score was calculated by multiplying the number of tiles shot by the percentage accuracy. Participants had three attempts, and the highest score was entered as the participant’s score for the visit.

#### 2.5.8 Safety and expectancy measures

The tolerability of capsule intake was evaluated through fortnightly questionnaires enquiring about adverse events and an interview at visit 2. The Global Assessment of Tolerability to Therapy (GATT) was also completed at visit 2, where participants indicated their tolerability to capsule intake on a 5-point scale ranging from poor to excellent.

As expectancies can influence treatment outcomes in placebo-controlled trials (35), participants completed the Clinical Trials Treatment Expectancies Scale (CTTES) at visit 1. The CTTES, a 6-item questionnaire, is a revision of the Stanford Expectations of Treatment Scale (36), with wording modified to reference clinical trials examining cognitive and sleep changes.

### 2.6 Sample size calculations

In previous trials investigating the cognitive-enhancing effects of health ingredients and nutraceuticals in healthy adults, effect sizes of 0.5 to 0.6 have been identified (37, 38). Therefore, an effect size of 0.55 was predicted. Assuming a power of 80% and a type one error rate (alpha) of 5%, the number of total participants required to find an effect is 84. Assuming a 10 to 15% dropout rate, it was planned to recruit 100 participants in total, which was hypothesised to give suitable power to find an effect compared to the placebo, even after dropouts.

### 2.7 Statistical analysis

For baseline data, an independent samples t-test was used to examine group differences for continuous variables, and a Pearson’s Chi-square test was used for the analysis of categorical data. Outcome analyses were conducted on the full analysis set (FAS) and per protocol set (PPS), where all participant data were retained in the originally assigned groups. Details of participants excluded from the PPS due to major protocol deviations are included in Supplementary Table 1. Generalised Linear Mixed Models (GLMM) were used to assess differences between intervention groups for all treatment outcomes, with intervention effects assessed via entry of the intervention group (placebo and Magtein^®^) x time interaction. The time points considered for the cognitive assessments were visit 1 (day −7) and visit 2 (day 42), and for the self-report questionnaires, days 0, 14, 28, and 42. Random intercepts were utilised in each model, and covariates of age, sex, and BMI were included.

The change in cognitive age was calculated using the NIH Toolbox Total Cognition Composite Score CSS. Composite CSS values were derived by averaging the test-level CSSs, calculating the standard error of the mean, and then normalising the composite scores against the NIH Toolbox version 3 age-unadjusted normative reference data. The resulting age-adjusted scores allowed each participant’s cognitive performance to be mapped to the age at which such performance would be expected, thereby quantifying whether cognition was younger or older than chronological age. An Analysis of Covariance (ANCOVA) was applied to evaluate treatment effects, with treatment as a fixed factor and baseline cognitive performance included as a covariate to control for initial baseline differences. To translate the cognitive improvements into a more intuitive metric, the equivalent “cognitive rejuvenation” effect was calculated by studying the change in “cognitive age” after Magtein^®^ treatment over placebo treatment (39). NIH toolbox contains CSS (age-unadjusted or raw score) for age groups from the version 3 normative sample for all NIH toolbox tests. Total cognition composite CSS peaks around age 20 and then declines approximately 3 points per decade (0.3 points per year) (40).

The CTTES positive and negative expectancy scores were also included for the self-report measures. For the Oura Ring scores, mean weekly scores were calculated for week 0 (days −7 to 0), week 1 (days 1–7), week 2 (days 8–14), week 3 (days 15–21), week 4 (days 22–28), week 5 (days 28–35), and week 6 (days 36–42). Due to the potential of increased variability in sleep routines on weekends, sleep data collected from Monday to Thursday evenings were only used for analysis. If the ring was not worn or recharged and a daily score was not obtained, mean scores were calculated using the available scores for the week. Where applicable, gamma (with log link function) and normal (with identity link function) target distributions were used. Appropriate covariance structures were used to model correlations between repeated time measurements in gamma models. Robust estimations were used to handle any violations of model assumptions. All data were analysed using SPSS (version 29; IBM, Armonk, NY). For all analyses, statistical significance was set at a two-tailed *p*-value of ≤ 0.05. Figures were generated using R version 4.4.3.

## 3 Results

### 3.1 Study population

As detailed in Figure 1, 156 people underwent a telephone screening and 100 people were randomised. The reasons for ineligibility were withdrawing consent after the telephone interview (*n* = 34), not meeting the eligibility criteria (*n* = 14) and extensive travel during the trial (*n* = 8).

### 3.2 Baseline questionnaire and demographic information

Baseline sociodemographic, clinical characteristics, and mean scores for the assessments at Visit 1 are included in Table 1. Analyses confirmed the groups were similar with no statistically significant differences between the groups.

**Table 1:** Baseline sociodemographic and clinical characteristics.

Baseline variable		Placebo (*n* = 50)	Magtein^®^ (*n* = 50)	*p*-value
Age (years)	Mean	36.82	37.07	0.849^a^
	SE	0.94	0.87	
Sex	Female	31	32	0.836^a^
	Male	19	18	
Height (m)	Mean	1.72	1.73	0.600^a^
	SE	0.01	0.01	
Weight (kg)	Mean	73.12	74.80	0.553^a^
	SE	2.25	1.72	
BMI (kg/m^2^)	Mean	24.62	24.98	0.597^a^
	SE	0.57	0.37	
Systolic blood pressure (mmHg)	Mean	118.00	118.34	0.886^a^
	SE	1.88	1.44	
Diastolic blood pressure (mmHg)	Mean	74.98	76.04	0.566^a^
	SE	1.36	1.24	
Marital status (n)	Single	19	18	0.836^b^
	Married/defector	31	32	
Educational level (n)	Secondary	14	17	0.203^b^
	Tertiary	18	23	
	Post-graduate	18	10	
IPAQ category (n)	Low	19	18	0.320^b^
	Moderate	20	26	
	High	11	6	
Occupation (n)	Unemployed	2	5	0.638^b^
	Services and sales worker	5	5	
	Professional	19	11	
	Elementary occupation	1	1	
	Unemployed	2	5	
	Clerical support worker	2	4	
	Craft and related trades worker	1	4	
	Manager	4	3	
	Student	7	7	
	Technicians and associated trades	9	10	
NIH Total Cognition Composite score (aged-adjusted standard score)	Mean	110.50	111.72	0.650^a^
	SE	1.94	1.85	
NIH Fluid Composite score (aged-adjusted standard score)	Mean	109.68	110.74	0.690^a^
	SE	1.70	2.04	
NIH Crystallised Composite score (aged-adjusted standard score)	Mean	107.14	108.36	0.644^a^
	SE	2.10	1.58	
Raven’s 2 (Standard Score)	Mean	110.38	108.50	0.381^a^
	SE	1.59	1.42	
Aim Trainer Score	Mean	56.81	56.07	0.684^a^
	SE	1.66	1.39	
PROMIS Sleep-Sleep Disturbance (T-Score) (Visit 1)	Mean	55.53	56.58	0.292^a^
	SE	0.68	0.72	
PROMIS Sleep-Sleep-Related Impairment (T-Score) (Visit 1)	Mean	57.70	58.30	0.617^a^
	SE	0.84	0.85	
RSQ (Visit 1)	Mean	47.72	45.06	0.359^a^
	SE	1.94	2.14	
WHO-5 (Visit 1)	Mean	13.62	13.22	0.553^a^
	SE	0.47	0.48	

a = Independent-Samples T-Test; b = Pearson Chi-Square Test; *p*-value = 2-sided.

### 3.3 Outcome measures

#### 3.3.1 NIH total cognition composite score

**Figure 2:**
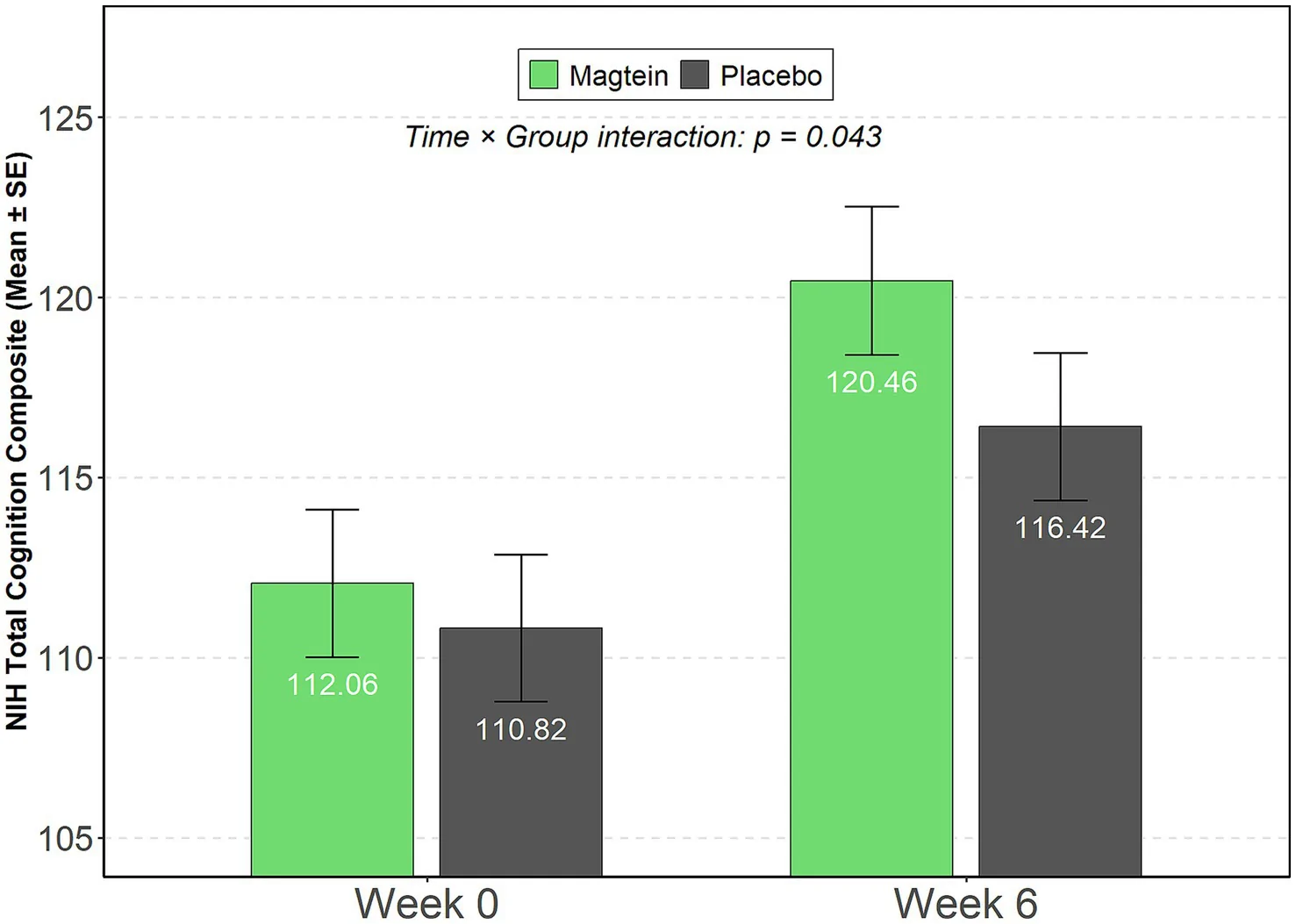
Change in NIH total cognition composite from week 0 (visit 1) to week 6 (visit 2) (FAS).

As demonstrated in Table 2 and Figure 2, based on the GLMM, there was a statistically significant time x group interaction in the Total Cognition Composite score (*p* = 0.043). In the Magtein^®^ group, the composite score increased by a mean of 8.40 points (95% CI: 6.48, 10.31, *p* < 0.001) and in the placebo group, it increased by a mean of 5.60 points (95% CI: 3.68, 7.51; *p* < 0.001). An analysis of the PPS revealed a more statistically significant group difference of 0.037 (Supplementary Table 2).

**Table 2:** Change in cognitive assessments (estimated marginal means) (Full analysis set).

Outcomes		Placebo (*n* = 50)				Magtein^®^ (*n* = 50)				*p*-value^b^
		Visit 1	Visit 2	Change from baseline	*p*-value^a^	Visit 1	Visit 2	Change from baseline	*p*-value^a^	
**NIH Total Cognition Composite**	**Mean**	**110.82**	**116.42**	**5.60**	**< 0.001**	**112.06**	**120.46**	**8.40**	**< 0.001**	**0.043**
	**SE**	**2.04**	**2.05**	**0.97**		**2.05**	**2.06**	**0.97**		
**NIH Fluid Composite**	**Mean**	**109.82**	**118.12**	**8.30**	**< 0.001**	**110.76**	**120.98**	**10.22**	**< 0.001**	**0.277**
	**SE**	**2.02**	**2.04**	**1.25**		**2.04**	**2.05**	**1.25**		
NIH Flanker Inhibitory Control & Attention	Mean	108.34	113.75	5.41	0.001	109.46	115.48	6.03	< 0.001	0.782
	SE	1.88	1.90	1.58		1.89	1.91	1.58		
NIH Dimensional Change Card Sort	Mean	101.15	107.34	6.19	< 0.001	106.93	113.77	6.84	< 0.001	0.781
	SE	2.43	2.45	1.66		2.44	2.46	1.66		
NIH Picture Sequence Memory	Mean	111.04	115.72	4.68	< 0.001	106.44	114.11	7.67	< 0.001	0.092
	SE	1.30	1.32	1.25		1.31	1.33	1.25		
NIH List Sorting Working Memory	Mean	108.94	110.32	1.38	0.367	106.22	112.22	6.00	< 0.001	0.033
	SE	1.62	1.64	1.52		1.63	1.65	1.52		
NIH Pattern Comparison tests	Mean	104.68	111.71	7.03	< 0.001	106.04	110.86	4.81	< 0.001	0.246
	SE	1.91	1.93	1.35		1.92	1.94	1.35		
**NIH Crystallised Composite**	**Mean**	**107.60**	**106.86**	**0.74**	**0.513**	**109.00**	**110.81**	**1.81**	**0.110**	**0.111**
	**SE**	**1.98**	**2.00**	**1.13**		**1.99**	**2.01**	**1.13**		
NIH Picture Vocabulary	Mean	101.76	102.35	0.59	0.647	103.50	105.83	2.33	0.072	0.341
	SE	2.03	2.05	1.29		2.04	2.06	1.29		
NIH Oral Reading Recognition	Mean	110.33	108.94	−1.39	0.331	111.34	112.25	0.92	0.521	0.254
	SE	1.96	1.98	1.42		1.97	1.99	1.42		
**Raven’s 2**	**Mean**	**110.69**	**111.76**	**1.07**	**0.535**	**109.10**	**110.02**	**0.92**	**0.592**	**0.953**
	**SE**	**1.65**	**1.67**	**1.72**		**1.65**	**1.68**	**1.72**		
**Aim Trainer**	**Mean**	**57.39**	**56.80**	**−0.59**	**0.667**	**56.79**	**60.38**	**3.59**	**0.009**	**0.031**
	**SE**	**1.34**	**1.36**	**1.36**		**1.35**	**1.37**	**1.36**		

Results (estimated means) are generated from generalised mixed-effects models adjusted for age, sex, and BMI.

a *p*-values are generated from repeated measures generalised mixed-effects models adjusted for age, sex, and BMI (time effects visit 1 and visit 2).

b *p*-values are generated from repeated measures generalised mixed-effects models for age, sex, and BMI (time x group interaction). Bold values represent composite and total scores.

#### 3.3.2 NIH total fluid composite score

**Figure 3:**
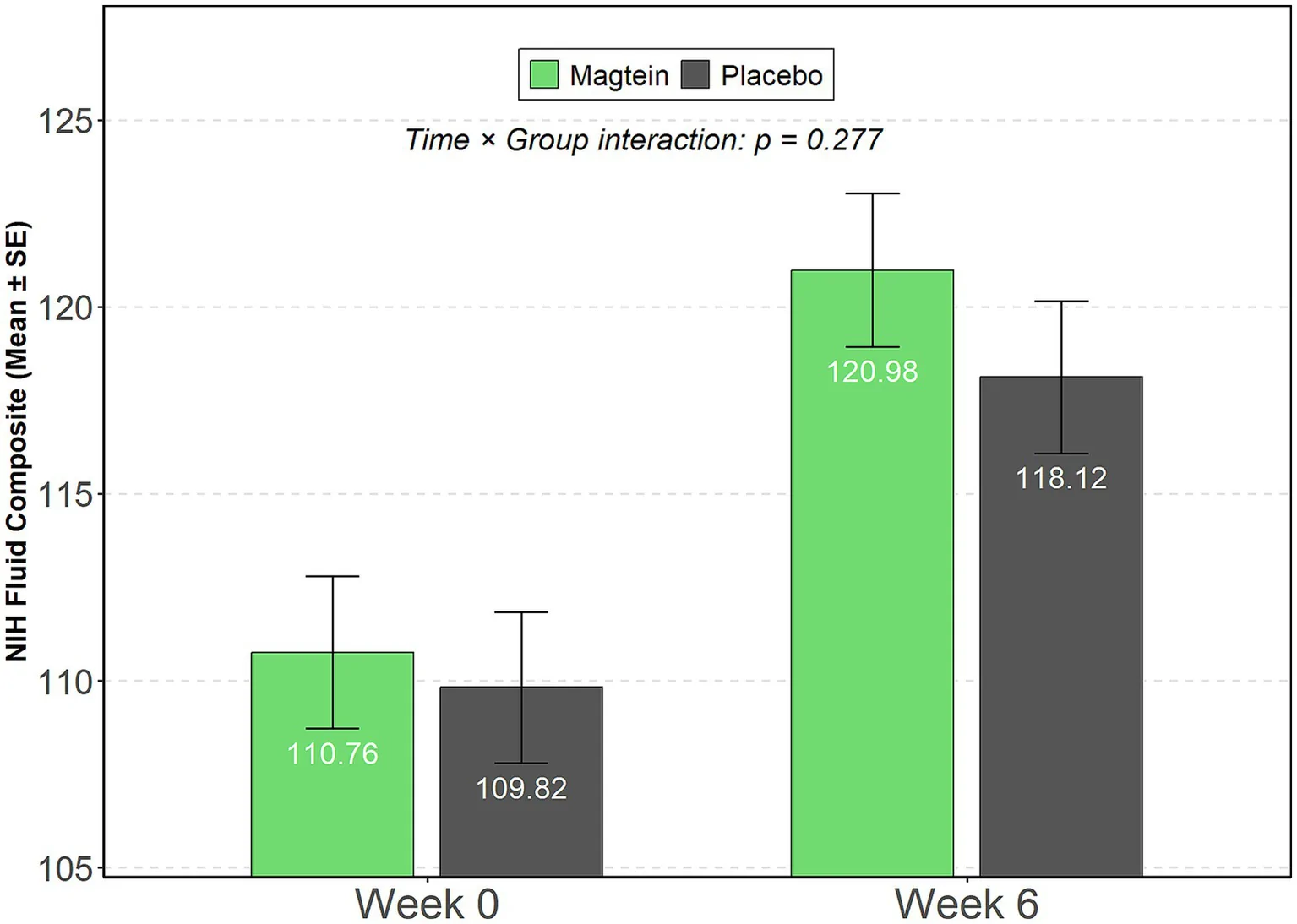
Change in NIH fluid composite from Week 0 (Visit 1) to Week 6 (Visit 2) (FAS).

As demonstrated in Table 2 and Figure 3, based on the GLMM, there was no statistically significant time x group interaction in the Fluid Composite score (*p* = 0.277). In the Magtein^®^ group, the composite score increased by a mean of 10.22 points (95% CI: 7.76, 12.69; *p* < 0.001) and in the placebo group, it increased by a mean of 8.30 points (95% CI: 5.84, 10.76; *p* < 0.001). An analysis of the PPS revealed similar findings (Supplementary Table 2).

#### 3.3.3 NIH total crystallised composite score

**Figure 4:**
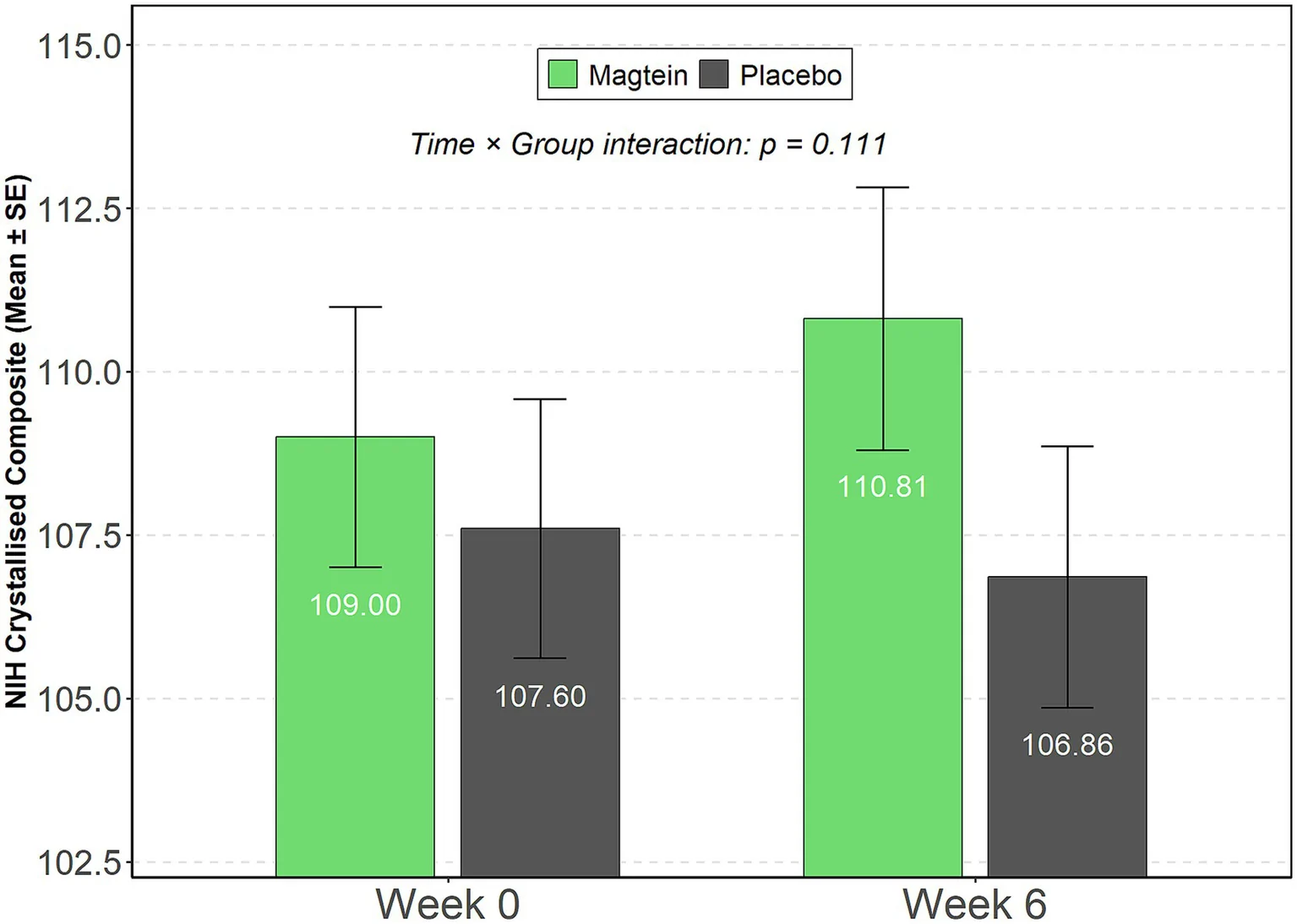
Change in NIH crystallized composite from week 0 (visit 1) to week 6 (visit 2) (FAS).

As demonstrated in Table 2 and Figure 4, based on the GLMM, there was no statistically significant time x group interaction in the Fluid Composite score (*p* = 0.111). There were no statistically significant changes in scores over time in the Magtein^®^ (95% CI: −0.41, 4.03, *p* = 0.110) and the placebo group (95% CI: −2.96, 1.48, *p* = 0.513). An analysis of the PPS revealed similar findings (Supplementary Table 2).

An examination of performance on individual cognitive tasks in the NIH cognitive toolbox (Table 2) revealed a statistically significant time x group interaction in the List Sorting Working Memory task (*p* = 0.033) and a strong trend of group differences in the Picture Sequence Memory task (*p* = 0.092). There were no other statistically significant group differences in changes in other cognitive tasks. An analysis of the PPS revealed a more significant change in the NIH List Sorting Working Memory, as shown in a smaller *p*-value of 0.010 (Supplementary Table 2).

#### 3.3.4 Raven’s 2 score

As demonstrated in Table 2, based on the GLMM, there was no statistically significant time x group interaction in Raven’s 2 total score (*p* = 0.953). There were no statistically significant changes in scores over time in the Magtein^®^ (95% CI: −2.47, 4.32, *p* = 0.592) and the placebo group (95% CI: −2.33, 4.46, *p* = 0.535). An analysis of the PPS revealed similar findings (Supplementary Table 2).

#### 3.3.5 Cognitive age

**Figure 5:**
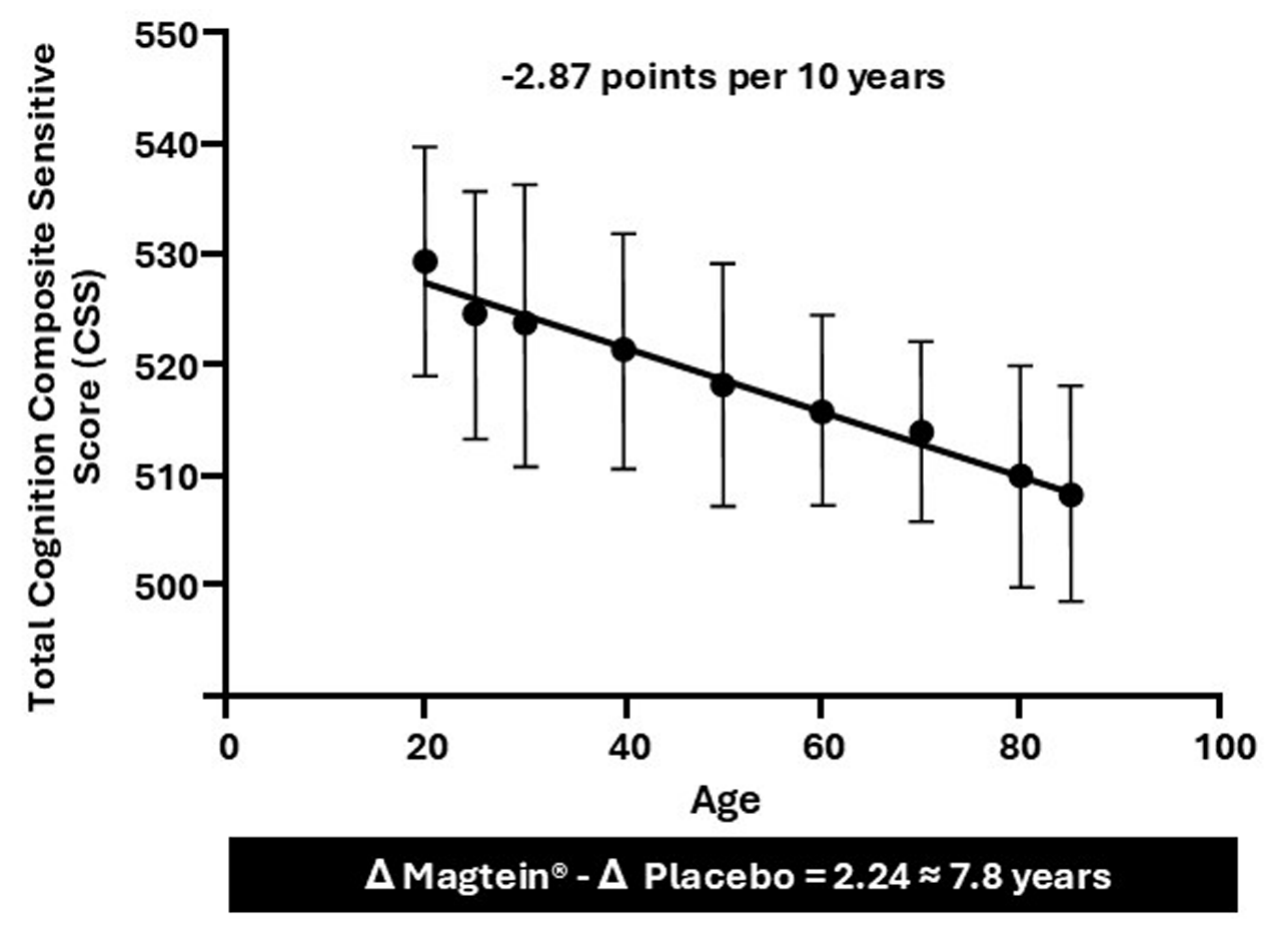
Created using data derived from Table A.1 CSS summary statistics for National Institutes of Health (NIH) toolbox tests, by age group (National Institutes of Health (NIH) Toolbox^®^ V3 Technical Manual) (40).

**Figure 6:**
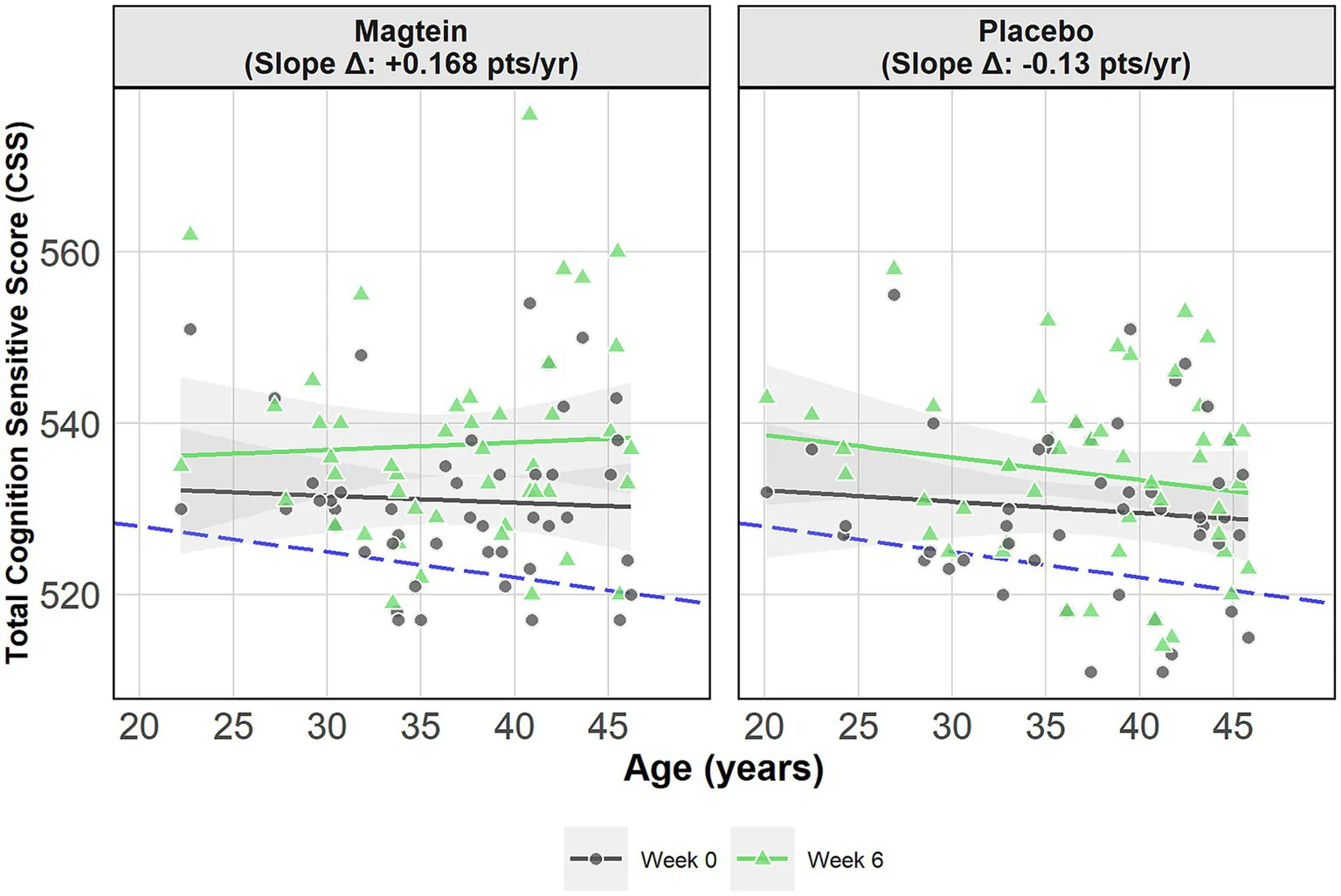
Linear regression of total cognition CSS versus participant age for each group, with the fitted equation and slope displayed. The blue dashed line shows the expected decline in the total cognition CSS score for the general population. The gradient change is calculated by subtracting the gradient at week 0 from the gradient at week 6, which is measured as the number of CSS scores that changed in a year.

The total CSS (age-unadjusted) was 537.07 for the Magtein^®^ group and 534.83 for the placebo at week 6, yielding a difference of 2.24 points (*p* = 0.041). Based on an estimated decline of 0.3 points per year from the age of 20 (40), a group difference of 2.24 points corresponds to an approximate 7.5-year difference in cognitive performance in the Magtein^®^ group compared to the placebo group (Figure 5). A further analysis of total cognition CSS versus age is demonstrated in Figure 6, which revealed divergent trajectories between the Magtein^®^ and placebo groups. While both groups displayed similar, age-related cognitive decline at baseline (Visit 1), their slopes diverged post-treatment. The Magtein^®^ group showed a significant improvement, with the slope reversing from −0.081 at baseline to +0.087 at Visit 2. However, the placebo group showed further decline, with its slope worsening from −0.13 to −0.26.

#### 3.3.6 Aim trainer score

As demonstrated in Table 2, based on the GLMM, there was a statistically significant time x group interaction in the score achieved on the Aim Trainer (*p* = 0.031). In the Magtein^®^ group, the score increased by a mean of 3.59 points (95% CI: 0.91, 6.28, 6.3% increase; *p* = 0.009), and in the placebo group, there was no statistically significant change (95% CI: −3.27, 2.10, *p* = 0.667). An analysis of the PPS revealed similar findings (Supplementary Table 2).

#### 3.3.7 PROMIS sleep disturbance score

As demonstrated in Table 3, based on the GLMM, there was no statistically significant time x group interaction in the PROMIS Sleep Disturbance score (*p* = 0.316). In the Magtein^®^ group, the score decreased by a mean of 5.62 points (95% CI: −3.99, −7.25; *p* < 0.001) and in the placebo group, it decreased by 3.81 points (95% CI: −2.17, −5.46; p < 0.001). An analysis of the PPS revealed similar findings (Supplementary Table 3). However, an exploratory analysis indicated that there were statistically significant group differences when a subset of participants with more severe sleep disturbances, represented by a T-score ≥ 56.5 (≥ 75th percentile) at screening were examined. As demonstrated in Table 4, based on the GLMM, there was a statistically significant time x group interaction in the PROMIS Sleep Disturbance score (*p* = 0.031). In the Magtein^®^ group, the score decreased by a mean of 7.68 points (95% CI: −5.54, −9.83; *p* < 0.001) and in the placebo group, it decreased by 3.84 points (95% CI: −1.19, −6.49; p < 0.001). An analysis of the PPS revealed similar findings, albeit with a greater statistical significance (*p*-value 0.009) (Supplementary Table 4).

**Table 3:** Change in self-report questionnaires (estimated marginal means) (full analysis set).

Outcomes		Placebo (*n* = 50)						Magtein^®^ (*n* = 50)						*p*-value^b^
		Day 0	Day 14	Day 28	Day 42	Change from baseline	*p*-value^a^	Day 0	Day 14	Day 28	Day 42	Change from baseline	*p*-value^a^	
PROMIS Sleep Disturbance (T-score)	Mean	55.52	51.85	51.55	51.71	−3.81	< 0.001	55.61	51.94	51.37	49.99	−5.62	< 0.001	0.316
	SE	0.83	0.78	0.78	0.79	0.84		0.85	0.81	0.79	0.77	0.83		
PROMIS Sleep-Related Impairment (T-score)	Mean	56.14	51.19	52.42	52.76	−3.38	0.001	57.79	53.01	51.77	51.44	−6.35	< 0.001	0.043
	SE	1.07	1.07	1.07	1.08	0.99		1.09	1.11	1.09	1.09	0.96		
RSQ	Mean	44.50	57.12	56.54	57.00	14.50	< 0.001	43.30	55.90	57.25	60.46	17.16	< 0.001	0.439
	SE	2.03	2.62	2.59	2.63	2.17		2.01	2.64	2.67	2.82	2.28		
WHO-5	Mean	12.63	15.09	14.85	15.06	2.42	< 0.001	12.70	15.14	15.39	16.12	3.42	< 0.001	0.436
	SE	0.48	0.58	0.57	0.58	0.45		0.49	0.60	0.60	0.63	0.48		

Results (estimated means) are generated from generalised mixed-effects models adjusted for age, sex, BMI, and CTTES positive and negative expectancies score.

a *p*-values are generated from repeated measures generalised mixed-effects models adjusted for age, sex, BMI, and CTTES positive and negative expectancies score (time effects day 0 and day 42).

b *p*-values are generated from repeated measures generalised mixed-effects models for age, sex, BMI, and CTTES positive and negative expectancies score (time x group interaction).

**Table 4:** Change in self-report questionnaires—selected sample (estimated marginal means) (full analysis set).

Outcomes		Placebo						Magtein^®^						*p*-value^b^
		Day 0	Day 14	Day 28	Day 42	Change from baseline	*p*-value^a^	Day 0	Day 14	Day 28	Day 42	Change from baseline	*p*-value^a^	
PROMIS Sleep Disturbance (T-score) (≥ 56.5; ≥ 75th percentile)	N	19						27						0.031
	Mean	58.16	52.95	54.08	54.32	−3.84	0.005	58.74	53.80	51.97	51.06	−7.68	< 0.001	
	SE	1.36	1.25	1.28	1.31	1.34		1.27	1.16	1.12	1.10	1.09		
PROMIS Sleep-Related Impairment (T-score) (≥ 56.5; ≥ 75^th^ percentile)	N	32						34						0.012
	Mean	58.00	53.29	54.91	55.48	−2.52	0.041	60.63	56.21	53.79	54.21	−6.43	< 0.001	
	SE	1.20	1.11	1.15	1.17	1.22		1.26	1.18	1.12	1.13	1.20		
RSQ (below sample median of 44)	N	26						29						0.640
	Mean	37.06	53.57	51.23	52.67	15.61	< 0.001	35.51	48.44	50.09	52.92	17.41	< 0.001	
	SE	2.49	3.60	3.44	3.58	2.91		2.32	3.19	3.30	3.49	2.77		

Results (estimated means) are generated from generalised mixed-effects models adjusted for age, sex, BMI, and CTTES positive and negative expectancies score.

a *p*-values are generated from repeated measures generalised mixed-effects models adjusted for age, sex, BMI, and CTTES positive and negative expectancies score (time effects day 0 and day 42).

b *p*-values are generated from repeated measures generalised mixed-effects models for age, sex, BMI, and CTTES positive and negative expectancies score (time x group interaction).

#### 3.3.8 PROMIS sleep-related impairment score

As demonstrated in Table 3, based on the GLMM, there was a statistically significant time x group interaction in the PROMIS Sleep-Related Impairment score (*p* = 0.043). In the Magtein^®^ group, the score decreased by a mean of 6.35 points (95% CI: −4.46, −8.24; *p* < 0.001) and in the placebo group, it decreased by 3.38 points (95% CI: −1.49, −5.26; *p* = 0.001). An analysis of the PPS revealed similar findings, albeit with greater statistical significance (Supplementary Table 3). Moreover, an exploratory analysis on a subset of participants with more severe sleep-related impairment, represented by a T-score ≥ 56.5 (≥ 75th percentile) at screening, demonstrated outcomes with greater statistical significance. As demonstrated in Table 4, based on the GLMM, there was a statistically significant time x group interaction in the PROMIS Sleep-Related Impairment score (*p* = 0.012). In the Magtein^®^ group, the score decreased by a mean of 6.43 points (95% CI: −4.07, −8.79; *p* < 0.001) and in the placebo group, it decreased by 2.52 points (95% CI: −0.11, −4.93; *p* = 0.041). An analysis of the PPS revealed similar findings (Supplementary Table 4).

#### 3.3.9 RSQ score

As demonstrated in Table 3, based on the GLMM, there was no statistically significant time x group interaction in the RSQ total score (*p* = 0.439). In the Magtein^®^ group, the RSQ score increased by a mean of 17.16 points (95% CI: 12.68, 21.64; *p* < 0.001) and in the placebo group, it increased by 14.50 points (95% CI: 8.23, 16.77; p < 0.001). An analysis of the PPS revealed similar findings (Supplementary Table 3). Moreover, an exploratory analysis on a subset of participants with more severe RSQ scores at screening, represented by an RSQ of < 44 (below the median score of the total sample) at screening revealed similar non-statistically significant group differences (Supplementary Table 4).

#### 3.3.10 WHO-5 total score

As demonstrated in Table 3, based on the GLMM, there was no statistically significant time x group interaction in the WHO-5 total score (*p* = 0.436). In the Magtein^®^ group, the WHO-5 score increased by a mean of 3.42 points (95% CI: 2.48, 14.36; p < 0.001) and in the placebo group, it increased by 2.42 points (95% CI: 1.54, 3.31; p < 0.001). An analysis of the PPS revealed similar findings (Supplementary Table 3).

#### 3.3.11 Oura Ring scores

Changes in outcomes measured by the Oura Ring are detailed in Table 5. There were no statistically significant group differences in changes in any sleep-related data. However, there were statistically significant between-group differences in changes in heart rate during sleep. Based on the GLMM, there was a statistically significant time x group interaction in the average heart rate during sleep (*p* = 0.030). In the Magtein^®^ group, the average heart rate decreased by 1.32 beats per minute (95% CI: −0.30, −2.33, *p* = 0.011) and in the placebo group, there was no statistically significant change (95% CI: −0.70, 1.28, *p* = 0.569). An analysis of the PPS revealed similar findings, albeit with a greater statistical significance (Supplementary Table 5). Moreover, there was a statistically significant time x group interaction in RMSSD (measure of HRV) (*p* = 0.036). In the Magtein® group, there was a non-significant increase in RMSSD of 1.45 ms (95% CI: 7–1.13, 4.04, *p* = 0.270), and in the placebo group, there was a non-significant decrease in RMSSD of 1.31 ms (95% CI: −3.53, 0.90, *p* = 0.245). An analysis of the PPS revealed similar statistically significant between-group differences (*p* = 0.046), with a near statistically significant increase in RMSSD of 2.49 ms over time in the Magtein^®^ group (95% CI: −0.15, 5.12, *p* = 0.065) (Supplementary Table 5).

**Table 5:** Change in Oura Ring measures (estimated marginal means) (full analysis set).

Outcomes		Placebo (*n* = 50)									Magtein^®^ (*n* = 50)									*p*-value^b^
		Week 0	Week 1	Week 2	Week 3	Week 4	Week 5	Week 6	Change from baseline	*p*-value^a^	Week 0	Week 1	Week 2	Week 3	Week 4	Week 5	Week 6	Change from baseline	*p*-value^a^	
Sleep Score	Mean	76.98	76.59	77.32	77.59	75.81	77.69	76.97	−0.01	0.989	74.74	74.76	73.77	74.77	73.56	75.27	73.40	−1.34	0.180	0.837
	SE	1.17	1.17	1.18	1.18	1.16	1.18	1.18	1.00		1.16	1.16	1.16	1.17	1.16	1.18	1.17	1.00		
Total duration of sleep period (minutes)	Mean	480.92	483.34	484.89	485.09	478.25	485.83	476.64	−4.28	0.610	479.19	477.47	459.62	476.63	461.80	480.53	460.70	−18.49	0.032	0.439
	SE	9.55	9.65	9.73	9.69	9.55	9.70	9.57	8.40		9.76	9.73	9.46	9.81	9.51	9.89	9.63	8.58		
Total sleep time (minutes)	Mean	414.90	413.11	417.25	419.68	410.62	419.58	411.56	−3.34	0.632	406.25	407.87	392.27	405.98	393.74	409.57	393.52	−12.72	0.071	0.461
	SE	7.89	7.91	8.02	8.03	7.86	8.03	7.91	6.98		7.93	7.96	7.73	8.01	7.77	8.08	7.88	7.03		
Total time awake (minutes)	Mean	65.56	69.77	67.49	65.40	67.33	66.19	64.89	−0.67	0.848	72.38	69.46	67.19	70.97	67.84	70.51	67.22	−5.16	0.188	0.599
	SE	4.17	4.46	4.34	4.18	4.31	4.23	4.17	3.51		4.73	4.54	4.43	4.68	4.47	4.65	4.49	3.92		
Light sleep (minutes)	Mean	249.72	247.61	253.82	252.02	246.29	249.29	246.89	2.83	0.590	243.96	246.72	233.48	249.49	238.58	248.87	234.72	9.24	0.079	0.080
	SE	6.08	6.06	6.24	6.17	6.03	6.10	6.07	5.24		6.09	6.16	5.88	6.29	6.02	6.28	6.01	5.26		
REM sleep (minutes)	Mean	88.73	89.04	88.41	90.29	89.75	92.00	89.23	0.51	0.841	87.32	89.12	85.28	83.62	83.87	87.64	85.88	−1.44	0.574	0.491
	SE	3.13	3.15	3.14	3.20	3.18	3.26	3.17	2.52		3.16	3.22	3.11	3.05	3.06	3.20	3.17	2.56		
Deep sleep (minutes)	Mean	76.15	76.02	74.97	77.19	74.18	78.15	74.80	−1.34	0.535	74.81	71.79	73.27	72.77	71.09	72.52	72.76	−2.04	0.354	0.776
	SE	2.47	2.48	2.46	2.52	2.42	2.55	2.45	2.17		2.49	2.39	2.46	2.45	2.39	2.44	2.48	2.20		
Sleep onset latency (minutes)	Mean	17.87	18.57	18.03	17.29	20.30	16.22	21.24	3.36	0.060	19.01	17.45	19.05	16.32	19.25	18.79	20.48	1.47	0.431	0.581
	SE	1.49	1.56	1.52	1.45	1.71	1.36	1.80	1.79		1.62	1.49	1.65	1.41	1.67	1.63	1.82	1.86		
Sleep efficiency (%)	Mean	86.36	85.57	86.38	86.59	86.07	86.55	86.66	0.30	0.634	84.94	85.60	85.50	85.65	85.55	85.63	85.60	0.67	0.306	0.785
	SE	0.74	0.74	0.75	0.74	0.74	0.74	0.75	0.63		0.74	0.75	0.76	0.76	0.76	0.76	0.77	0.65		
Wake up count (n)	Mean	5.58	5.86	5.27	5.57	5.62	5.66	5.38	−0.20	0.402	5.31	5.26	4.96	5.25	5.30	5.28	5.14	−0.17	0.469	0.969
	SE	0.28	0.29	0.27	0.28	0.28	0.28	0.27	0.24		0.27	0.27	0.26	0.27	0.28	0.27	0.27	0.24		
Average heart rate (bpm)	Mean	63.17	63.32	63.06	63.31	62.65	63.31	63.46	0.29	0.569	62.96	62.61	62.00	63.41	63.21	62.62	61.65	−1.32	0.011	0.030
	SE	1.11	1.11	1.11	1.11	1.10	1.11	1.12	0.50		1.13	1.13	1.12	1.15	1.14	1.13	1.12	0.52		
Heart rate variability -RMSSD (ms)	Mean	41.15	40.73	41.12	41.19	42.17	41.55	39.84	−1.31	0.245	44.62	45.71	47.20	44.41	43.14	46.50	46.07	1.45	0.270	0.036
	SE	2.76	2.74	2.76	2.77	2.83	2.79	2.68	1.13		3.08	3.15	3.26	3.07	2.98	3.21	3.19	1.32		

Results (estimated means) are generated from generalised mixed-effects models adjusted for age, sex, and BMI.

a *p*-values are generated from repeated measures generalised mixed-effects models adjusted for age, sex, and BMI (time effects week 0 and week 6).

b *p*-values are generated from repeated measures generalised mixed-effects models for age, sex, and BMI (time x group interaction).

As an exploratory post-hoc analysis, the correlation between baseline (week 0) Oura Ring measures and baseline scores on self-report questionnaires and cognitive tests was examined. As detailed in Supplementary Table 6, the average resting heart rate during sleep was the only Oura Ring measure that was significantly correlated with all self-report questionnaire scores at baseline, whereby a lower heart rate was associated with better self-reported sleep quality, restorative sleep, and emotional wellbeing.

### 3.4 Intake of supplements

Interventional product bottles with remaining capsules were returned by participants on visit 2. Based on a capsule count of returned capsules at visit 2, 92% (*n* = 88) of participants who completed the study took over 80% of their capsules.

### 3.5 Efficacy of participant blinding

To assess the effectiveness of condition concealment during the trial, participants predicted their condition allocation (i.e., placebo, magnesium, or unsure) at the end of the study. Overall group concealment was high, as 54% of participants in the placebo group and 62% of participants in the Magtein^®^ group were unsure or incorrectly guessed treatment allocation.

### 3.6 Adverse reactions and treatment discontinuation

Table 6 details the adverse events possibly or probably related to the study interventions. There were no significant between-group differences in the frequency of self-reported treatment-related adverse events. In the placebo group, 8.0% (*n* = 4) of participants experienced a treatment-related adverse event, and in the Magtein^®^ group, 10% (*n* = 5) of participants experienced a treatment-related adverse event. The GATT completed at visit 2 demonstrated that in the Magtein^®^ group, 98% of participants reported good or excellent tolerability to capsule intake (compared to 100% in the placebo group). One person in the Magtein^®^ group reported moderate tolerability. A total of 4 people discontinued the study (2 in each group). No participant in the Magtein^®^ group discontinued due to a treatment-related adverse event, although one person in the placebo group discontinued due to gastrointestinal symptoms believed to be associated with capsule intake.

**Table 6:** Possibly or probably related adverse events by class and term.

AE Class	Diagnosis or symptom	Placebo (*n* = 50)	MgT (*n* = 50)
Gastrointestinal	**Number of participants**	**2 (4.0%)**	**2 (4.0%)**
	Increased bowel movements/ loose stools	1 (2.0%)	2 (4.0%)
	Constipation	1 (2.0%)	0 (0.0%)
	Abdominal pain	2 (4.0%)	2 (4.0%)
Neurological	**Number of participants**	**1 (2.0%)**	**2 (4.0%)**
	Headaches	1 (2.0%)	2 (4.0%)
Dermatological	**Number of participants**	**0 (0.0%)**	**1 (2.0%)**
	Skin rash	0 (0.0%)	1 (2.0%)
**Number of participants experiencing no treatment-related adverse events**		**46 (92%)**	**45 (90%)**

*Some participants experienced more than one treatment-related adverse event. Bold values represent total number/ percentage of participants per AE class.

## 4 Discussion

In this 6-week, randomised, double-blind, placebo-controlled study, the effect of Magtein^®^ supplementation on cognitive performance and sleep was examined in adults aged 18 to 45 years with self-reported dissatisfied sleep. Based on the results of the primary outcome measure (NIH Toolbox Total Cognition Composite), Magtein^®^ at a dose of 1 g twice daily was associated with a greater improvement in overall cognitive performance compared to the placebo. These findings are consistent with, and extend on a previous clinical trial on older adults aged 50 to 70 years, which demonstrated Magtein^®^ significantly improved composite cognitive scores across four domains (executive function, working memory, attention, and episodic memory), where a large treatment effect size was identified (Cohen’s d = 0.91 at 12 weeks). Similarly, in a trial on healthy adults aged 18 to 65 years, robust gains across all five subtests of the Clinical Memory Test (directed memory, paired-association learning, free recall, recognition, and portrait-feature memory) were observed, as well as significant increases in the overall memory quotient after 30 days of supplementation with Magtein^®^ (19). Collectively, these studies provide substantiation through three independent assessment tools (a composite cognitive z-score battery, the Clinical Memory Test, and the NIH Toolbox Total Cognition Composite) that Magtein^®^ may improve cognitive performance. Moreover, Magtein^®^ has enhanced cognitive performance across diverse age groups (18 to 70 years) with supplementation periods ranging from 4 to 12 weeks.

The NIH Toolbox comprises tasks designed to assess key cognitive domains relevant to daily functioning, including executive function, episodic memory, attention, processing speed, and language (40, 41). Among these, the List Sorting Working Memory (LSWM) test assesses the capacity to temporarily store, manipulate, and reorder information, a core aspect of executive function dependent on prefrontal–parietal circuitry (41). The Picture Sequence Memory Test (PSMT), in contrast, measures episodic memory through the recall of ordered visual events, relying heavily on hippocampal and medial temporal lobe networks (41, 42). In this study, participants receiving Magtein^®^ showed statistically significant greater gains on the LSWM and a strong trend toward greater improvement on the PSMT. This pattern suggests that Magtein^®^ may exert its strongest effects on cognitive domains sensitive to synaptic plasticity and hippocampal function, including working memory and episodic memory, rather than on more crystallised or less plastic domains. These findings are consistent with the established biological role of magnesium in supporting synaptic density and neural connectivity, particularly within brain regions subserving memory and executive control (14, 15, 19).

Hand–eye coordination is a critical skill that reflects the brain’s ability to process visual information rapidly and translate it into precise motor actions. Digital aim trainers, such as the 3D Aim Trainer, provide an objective and repeatable method to measure this coordination by tracking speed, accuracy, and consistency of responses (43). In e-sport video games, players must employ diverse control strategies to react rapidly to fast-moving visual and auditory stimuli, while also maintaining the flexibility to adapt their decisions to an ever-changing context (44). In this study, the 3D Aim Trainer was employed as an outcome measure to assess changes in hand-eye coordination, visuo-motor skills, and reaction time. To our knowledge, this is the first investigation examining the effects of Magtein^®^, or any other form of magnesium, on visuo-motor skills. Results from the Aim Trainer confirmed Magtein^®^ was associated with significantly greater improvements in performance on this task compared to the placebo. This suggests Magtein^®^ supplementation can enhance visuo-motor reaction time and hand–eye coordination. While further research is required, utilising a more comprehensive battery of tools assessing visuo-motor performance, these improvements may translate into real-life benefits such as faster responses while driving, improved performance in sports or gaming, and greater efficiency in everyday tasks that rely on quick visuo-motor skills (44, 45).

Despite improvements in the previously mentioned cognitive tasks, there were no changes in the Raven’s 2 task performance, a widely recognised nonverbal measure of fluid intelligence that requires abstract reasoning and pattern recognition, independent of acquired knowledge. Raven’s tasks are less directly reliant on hippocampal or prefrontal plasticity and more reflective of stable reasoning ability, which may explain the absence of measurable effects within the six-week intervention (46–48).

Using the NIH Total Cognition Composite score, changes in cognitive age were calculated, whereby a younger cognitive age indicates better-than-expected brain performance for one’s age, while an older cognitive age suggests an accelerated decline. This concept has become increasingly important in neuroscience and clinical research, as it provides a measurable way to assess interventions, such as nutritional supplementation, that may help preserve or even improve brain function (27, 49). Analyses conducted after 6 weeks of supplementation revealed that compared to the placebo, participants supplemented with Magtein^®^ experienced 7.5 years of cognitive rejuvenation, with exploratory analyses demonstrating larger gains in older participants. These results are consistent with findings from a 12-week, randomised-controlled trial on adults aged 50 to 70 years supplemented with Magtein^®^, where improvements corresponded to a reversal of cognitive age by approximately 9 years (19). While further research is required, these results suggest Magtein^®^ may support healthy aging of cognitive abilities, with greater efficacy more likely in older participants. It is important to note that individuals in the current study were relatively young, with a mean age of 37 years and cognitive performances at baseline already above age-expected norms (standard score of approximately 110, which is one standard deviation above the mean). Therefore, Magtein^®^ still has the potential to support healthy aging of cognitive abilities in individuals with adequate cognitive function (19).

An examination of sleep-related changes over time demonstrated that Magtein^®^ supplementation was associated with greater improvements in self-reported sleep-related impairment compared to the placebo. However, there were no group differences in changes in self-reported sleep disturbance, restorative sleep, or in measures obtained through the Oura Ring. An exploratory analysis revealed that participants with PROMIS Sleep scores above the 75th percentile experienced larger improvements in both sleep-related impairment and sleep disturbances. This suggests Magtein^®^ supplementation has greater efficacy in people with more significant sleep-related difficulties. However, as previously mentioned, there were no group differences based on the RSQ, a self-report measure of restorative sleep. A significant placebo response was observed on the RSQ, which persisted until at least day 28. Some group differentiation emerged on day 42, but this was not statistically significant. These subjective sleep-related improvements are generally consistent with results from a recent randomised, placebo-controlled clinical trial in middle-aged adults aged 35 to 55 years, where 3 weeks of Magtein^®^ supplementation significantly improved sleep quality compared to placebo. The subjective sleep results from this study indicate that the benefits of Magtein^®^ are most pronounced in populations with greater baseline sleep dissatisfaction, while improvements are less detectable in individuals without such disturbances, possibly due to a ceiling effect (50).

There were no statistically significant changes in Oura Ring’s sleep-related data over time for any treatment group (placebo or Magtein^®^). This contrasts with the significant changes in self-report measures over time in both the placebo and Magtein^®^ conditions. Differences in subjective and objective measures of sleep are commonly identified in research (51, 52). Moreover, while the Oura Ring has been demonstrated as a sound measure of sleep duration and sleep efficiency, other sleep measures, such as actigraphy and polysomnography, could be considered (34, 53). It is important to note that at baseline, the mean total sleep time for the whole population was 6 h and 53 min, and sleep efficiency was 86% (54, 55). These are considered within healthy levels, suggesting sleep disturbances, from an objective perspective, were minimal in the recruited population. Thus, identifying changes in sleep using the Oura ring in this study may, therefore, be difficult due to ceiling effects.

During sleep, heart rate indicators were measured by the Oura Ring. This included average resting heart rate and HRV during sleep. Compared to the placebo, there was a significant decrease in heart rate during sleep, and an increase in RMSSD, a measure of HRV. These results suggest that Magtein^®^ may influence the activity of the autonomic nervous system during sleep, and to our knowledge, this is the first study to demonstrate an effect of dietary magnesium supplementation on HRV and resting heart rate during sleep. RMSSD is an indicator of the autonomic nervous system’s parasympathetic branch (56), and an increase in RMSSD and a reduction in heart rate suggest Magtein^®^ may increase parasympathetic activity. An increase in parasympathetic activity is associated with increased relaxation and may contribute to improved sleep quality (57). An increase in RMSSD has also been associated with better cognitive performance (58, 59). HRV is also a well-established biomarker of stress and autonomic balance. Under stress, HRV and RMSSD decrease due to reduced parasympathetic activity, while relaxation increases both HRV and vagal tone. Across studies, higher RMSSD consistently indicates lower stress and better autonomic regulation. This supports the notion that the observed increase in RMSSD with Magtein^®^ reflects not only improved sleep physiology but also enhanced stress resilience and autonomic recovery (60, 61). Additionally, magnesium plays a well-established role in cardiovascular regulation, influencing both vascular tone and autonomic balance (62). By supporting parasympathetic activity, magnesium has been shown to reduce daytime resting heart rate and improve HRV, both of which are strong indicators of cardiovascular health (63). The observation that Magtein^®^ supplementation decreased heart rate during sleep while increasing RMSSD suggests that Magtein^®^ supplementation may also extend to beneficial effects on cardiac autonomic control. Magtein^®^ has been shown to effectively cross the blood–brain barrier (14, 64) and, therefore, has the potential to be delivered into cardiac tissue. This is a potential area of investigation in future trials.

Interestingly, in a post-hoc exploratory analysis, average heart rate during sleep at baseline was the only Oura Ring measure that was significantly correlated with baseline scores on all the self-report questionnaires (PROMIS Sleep, RSQ, and WHO-5), whereby a lower heart rate was associated with better sleep quality, restorative sleep, and better emotional wellbeing. This suggests that a lower heart rate during sleep, as measured by the Oura Ring, is significantly correlated with subjective sleep quality and emotional wellbeing.

### 4.1 Strengths, limitations, and directions for future research

Although there were some positive findings identified in the study, several recommendations for further research are offered. While improvements were identified in overall cognitive function, administering a more comprehensive battery of cognitive tasks that specifically measure working memory, episodic memory, visuo-motor performance, and other cognitive domains will be important in future trials. In this study, the effects of Magtein^®^ on cognition were examined in young-to-middle-aged adults. An examination of the effects of Magtein^®^ in older populations and in people experiencing cognitive impairments will also be important. In a double-blind, placebo-controlled study, improvements in general cognitive function were demonstrated in healthy adults aged 18 to 65 years after 30 days of supplementation with Magtein^®^ (17). Moreover, in a randomised, double-blind, placebo-controlled study on older adults aged 50 to 70 years, overall cognitive ability improved after 12 weeks of supplementation (19). In an open-label study, improvements in cognitive function, as measured by the Mini-Mental State Examination, were observed after 8 weeks in people with mild-to-moderate Alzheimer’s disease (65). In another open-label, 12-week study on adults with ADHD, some improvements in cognitive function were also observed (18). However, the robustness of conclusions from these studies is negatively impacted by open-label designs, small sample sizes, and, in most cases, the delivery of Magtein^®^ in combination with additional nutrients that could also affect cognitive function. Furthermore, while the results of this study provide support for the cognitive and sleep-enhancing benefits of Magtein^®^ supplementation, the findings should be extended cautiously to other forms of magnesium. All studies examining the effects of magnesium supplementation as a stand-alone intervention on cognitive function have been conducted using Magtein^®^ (17–19), so further research is required to determine if such cognitive benefits apply to other forms of magnesium. This has particular pertinence as Magtein^®^ has been shown through animal and *in vitro* studies to increase brain magnesium concentrations, which has not been demonstrated convincingly with other forms of magnesium (13–16). Sleep-related benefits have been identified with Magtein^®^ (50) and other magnesium forms (24), although there have been no studies directly comparing the sleep-related effects of different magnesium forms. As an improvement in deep sleep was identified in a recent study on Magtein^®^ (50), it will be important to investigate in future trials if different magnesium forms have varying effects on sleep stages. Magtein^®^ has been demonstrated in previous trials to have good tolerance with a low prevalence of gastrointestinal-related disturbances. Typically, the most common adverse reaction associated with magnesium supplementation is gastrointestinal disturbances such as diarrhoea or loose stools, particularly at higher doses (66). However, this was not observed in the current study or other trials conducted on Magtein^®^.

Findings from meta-analyses have demonstrated that blood concentrations of magnesium are positively associated with cognitive performance (67) and sleep quality (24). Therefore, investigating the effects of Magtein^®^ supplementation on cognition and sleep in individuals with magnesium deficiency or low blood magnesium concentrations will be worthwhile, as greater therapeutic efficacy may be evident in such populations. The results of this study demonstrated improvements in subjective sleep after 6 weeks of treatment. Therefore, studies of longer duration will be beneficial to understand the effects of Magtein^®^ supplementation on sleep over a longer period. The use of additional objective sleep measures will also be useful to further examine the effects of Magtein^®^ on sleep architecture. Sleep diaries can also be utilised to better identify sleep onset and wake times. Moreover, the recruitment of participants with more severe sleep-related disturbances and/or people with diagnosed insomnia will be useful.

The results obtained from the Oura Ring suggest Magtein^®^ supplementation may increase parasympathetic activity. Further trials will be important to validate and extend these findings. In particular, the use of experimental stress models may help elucidate the effects of Magtein^®^ on the stress response, whereby objective and subjective measures of stress are examined. Examples of experimental stress models include the Maastricht Stress Test and the Trier Social Stress Test. A further investigation into the effects of Magtein^®^ on HRV and other objective stress-related measures, such as cortisol and salivary amylase, may also be useful. An interesting and unique exploratory finding was that a lower resting heart rate during sleep was significantly associated with subjective sleep quality, restorative sleep, and emotional wellbeing. However, no other Oura Ring measures were significantly associated with subjective measures. This observation requires investigation in further studies as it presents a potential target of intervention that may improve subjective changes in wellbeing over time. Moreover, as already mentioned, resting heart rate was lowered by Magtein^®^ supplementation. Although not investigated in this study, the anxiolytic and mood-enhancing effects of Magtein^®^ will be of merit as a strong relationship between sleep quality, cognitive function, and emotional wellbeing exists (68). Magnesium has shown promise as an anxiolytic agent (69), and in a small study on Magtein^®^, some anxiolytic and stress-lowering effects were demonstrated (70).

## 5 Conclusion

In summary, the results of this study demonstrate that magnesium L-threonate (Magtein^®^) supplementation for 6 weeks was associated with improvements in cognitive performance in young-to-middle-aged adults. Moreover, improvements in hand-eye coordination and reaction time were observed. Improvements in subjective sleep were demonstrated, confirmed with a subjective sleep questionnaire (PROMIS Sleep). No changes in sleep parameters as measured by the Oura Ring were demonstrated, although a reduction in resting heart rate and an increase in HRV during sleep were demonstrated. This suggests Magtein^®^ may increase parasympathetic activity. This study builds on previous clinical trials, which have shown a benefit of Magtein® supplementation in various populations on overall cognition.

## Data Availability

The raw data supporting the conclusions of this article will be made available by the authors, without undue reservation.

## Footnotes

1
www.3daimtrainer.com
