# Correction: The effects of magnesium L-threonate (Magtein^®^) on cognitive performance and sleep quality in adults: a randomised, double-blind, placebo-controlled trial

**DOI:** 10.3389/fnut.2026.2010644

**Published:** 2026-10-15

**Authors:** Adrian L. Lopresti, Stephen J. Smith

**Affiliations:** 1 Clinical Research Australia, Perth, WA, Australia; 2 College of Science, Health, Engineering and Education, Murdoch University, Perth, WA, Australia

**Keywords:** brain aging, clinical trial, cognition, magnesium L-threonate, Magtein^®^, memory, NIH toolbox, sleep

An error was identified in Section *2.7 Statistical analysis*. The article incorrectly stated that the critical p-value was one-tailed. All statistical analyses were conducted using two-tailed tests. This was a wording error only and does not affect any of the reported *p*-values, results, interpretations, or conclusions.

A correction has been made to the section **Materials and methods**, *2.7 Statistical analysis*, Paragraph 3:

“The CTTES positive and negative expectancy scores were also included for the self-report measures. For the Oura Ring scores, mean weekly scores were calculated for week 0 (days −7 to 0), week 1 (days 1–7), week 2 (days 8–14), week 3 (days 15–21), week 4 (days 22–28), week 5 (days 28–35), and week 6 (days 36–42). Due to the potential of increased variability in sleep routines on weekends, sleep data collected from Monday to Thursday evenings were only used for analysis. If the ring was not worn or recharged and a daily score was not obtained, mean scores were calculated using the available scores for the week. Where applicable, gamma (with log link function) and normal (with identity link function) target distributions were used. Appropriate covariance structures were used to model correlations between repeated time measurements in gamma models. Robust estimations were used to handle any violations of model assumptions. All data were analysed using SPSS (version 29; IBM, Armonk, NY). For all analyses, statistical significance was set at a two-tailed *p*-value of ≤ 0.05. Figures were generated using R version 4.4.3.”

The original version of this article has been updated.

