# Supplementary material for: The effects of magnesium L-threonate (Magtein®) on cognitive performance and sleep quality in adults: a randomised, double-blind, placebo-controlled trial
Source: Front Nutr. 2026 Jan 12;12:1729164. doi: 10.3389/fnut.2025.1729164 (PMC12832366; doi:10.3389/fnut.2025.1729164)
Supplement: Supplementary file 1 [file Table_1.docx]

Supplementary Material

**An examination into the effects of magnesium L-threonate (Magtein®) on cognitive performance and sleep quality in adults experiencing dissatisfied sleep: a randomised, double-blind, placebo-controlled trial**

**Author names:**

^1,2^ Adrian L Lopresti - ORCID iD: 0000-0002-6409-7839

^1^ Stephen J Smith - ORCID iD: 0000-0002-3875-4815

**Author affiliations:**

^1^Clinical Research Australia, Perth, Western Australia, 6023, Australia

^2^College of Science, Health, Engineering and Education, Murdoch University, Perth, Western Australia, 6150, Australia

**Corresponding author:** Adrian Lopresti, Clinical Research Australia, 38 Arnisdale Road Duncraig, Western Australia 6023, [adrian@clinicalresearch.com.au](mailto:adrian@clinicalresearch.com.au)

**Supplementary Table 1. Details of participants with major protocol deviations and excluded from the Per Protocol set**

| **Number** | **Details of Deviation** |
| --- | --- |
| **Magtein group** | |
| 1 | Withdrew from the study |
| 2 | Withdrew from the study |
| 3 | Took 73% of IP |
| 4 | Took 75% of IP |
| 5 | Took 65% of IP |
| 6 | Took 70% of IP |
| 7 | Completed Raven's 2 at visit 2 in 5 minutes |
| 8 | Completed Raven's 2 at visit 2 in 6 minutes |
| 9 | Commenced prohibited concomitant medications:  Metronidazole, amoxicillin, codeine (infected wisdom tooth), Vortioxetine (depression) |
| 10 | Commenced prohibited concomitant medications:  Naproxen, Pregabalin (Disc bulge/ back pain), Diazepam (insomnia), Pantoprazole (Reflux) |
| **Placebo group** | |
| 1 | Withdrew from the study |
| 2 | Withdrew from the study |
| 3 | Took double dose for the first 2 weeks |
| 4 | Took 69% of IP |
| 5 | Took 57% of IP |
| 6 | Took 76% of IP |
| 7 | Took 73% of IP |
| 8 | Completed Raven's 2 at visit 2 in 6 minutes |
| 9 | Completed Raven's 2 at visit 1 in 5 minutes |

**Supplementary Table 2. Change in Cognitive Assessments (estimated marginal means) (Per Protocol Set)**

|  |  | Placebo (n=41) | | | | Magtein^®^ (n=40) | | | | p-value^b^ |
| --- | --- | --- | --- | --- | --- | --- | --- | --- | --- | --- |
|  |  | Visit 1 | Visit 2 | Change from baseline | p-value^a^ | Visit 1 | Visit 2 | Change from baseline | p-value^a^ |  |
| **NIH Total Cognition Composite** | **Mean** | **110.30** | **116.07** | **5.78** | **< 0.001** | **111.00** | **120.08** | **9.08** | **< 0.001** | **0.037** |
|  | **SE** | **2.24** | **2.24** | **1.10** |  | **2.28** | **2.28** | **1.12** |  |  |
| **NIH Fluid Composite** | **Mean** | **109.60** | **118.26** | **8.71** | **< 0.001** | **109.6** | **120.88** | **11.25** | **< 0.001** | **0.207** |
|  | **SE** | **2.21** | **2.21** | **1.41** |  | **2.24** | **2.24** | **1.43** |  |  |
| NIH Flanker Inhibitory Control & Attention | Mean | 108.63 | 113.93 | 5.29 | 0.003 | 109.14 | 115.12 | 5.97 | 0.001 | 0.783 |
|  | SE | 2.04 | 2.04 | 1.74 |  | 2.07 | 2.07 | 1.76 |  |  |
| NIH Dimensional Change Card Sort | Mean | 100.67 | 107.06 | 6.39 | 0.001 | 106.42 | 113.87 | 7.45 | < 0.001 | 0.683 |
|  | SE | 2.71 | 2.71 | 1.82 |  | 2.75 | 2.75 | 1.84 |  |  |
| NIH Picture Sequence Memory | Mean | 110.56 | 115.78 | 5.22 | < 0.001 | 105.90 | 114.20 | 8.30 | < 0.001 | 0.092 |
|  | SE | 1.37 | 1.37 | 1.28 |  | 1.40 | 1.40 | 1.29 |  |  |
| NIH List Sorting Working Memory | Mean | 109.72 | 110.81 | 1.10 | 0.507 | 106.25 | 113.50 | 7.25 | < 0.001 | 0.010 |
|  | SE | 1.81 | 1.81 | 1.65 |  | 1.84 | 1.84 | 1.67 |  |  |
| NIH Pattern Comparison tests | Mean | 104.07 | 111.83 | 7.76 | < 0.001 | 104.40 | 110.13 | 5.73 | < 0.001 | 0.315 |
|  | SE | 2.07 | 2.07 | 1.41 |  | 2.10 | 2.10 | 1.43 |  |  |
| **NIH Crystallised Composite** | **Mean** | **106.9** | **106.06** | **-0.83** | **0.520** | **108.4** | **110.31** | **1.88** | **0.152** | **0.141** |
|  | **SE** | **2.20** | **2.20** | **1.29** |  | **2.23** | **2.23** | **1.30** |  |  |
| NIH Picture Vocabulary | Mean | 101.00 | 101.97 | 0.98 | 0.513 | 102.20 | 104.70 | 2.50 | 0.099 | 0.473 |
|  | SE | 2.25 | 2.25 | 1.49 |  | 2.28 | 2.28 | 1.51 |  |  |
| NIH Oral Reading Recognition | Mean | 109.69 | 108.01 | -1.68 | 0.298 | 111.54 | 112.34 | 0.80 | 0.625 | 0.281 |
|  | SE | 2.13 | 2.13 | 1.61 |  | 2.16 | 2.16 | 1.63 |  |  |
| **Raven's 2** | **Mean** | **110.99** | **111.28** | **0.29** | **0.870** | **108.83** | **110.23** | **1.40** | **0.440** | **0.664** |
|  | **SE** | **1.77** | **1.77** | **1.79** |  | **1.79** | **1.79** | **1.81** |  |  |
| **Aim Trainer** | **Mean** | **57.22** | **56.92** | **-0.30** | **0.849** | **56.43** | **60.72** | **4.29** | **0.008** | **0.042** |
|  | **SE** | **1.52** | **1.52** | **1.57** |  | **1.54** | **1.54** | **1.59** |  |  |

Results (estimated means) are generated from generalised mixed-effects models adjusted for age, sex, and BMI. ^a^P-values are generated from repeated measures generalised mixed-effects models adjusted for age, sex, and BMI (time effects visit 1 and visit 2). ^b^P-values are generated from repeated measures generalised mixed-effects models for age, sex, and BMI (time x group interaction).

**Supplementary Table 3. Change in Self-Report Questionnaires (estimated marginal means) (Per Protocol Set)**

|  | | Placebo (n=41) | | | | | | | Magtein^®^ (n=40) | | | | | | p-value^b^ |
| --- | --- | --- | --- | --- | --- | --- | --- | --- | --- | --- | --- | --- | --- | --- | --- |
|  |  | Day 0 | Day 14 | Day 28 | Day 42 | Change from baseline | p-value^a^ | Day 0 | | Day 14 | Day 28 | Day 42 | Change from baseline | p-value^a^ |  |
| PROMIS Sleep Disturbance (T-score) | Mean | 55.75 | 52.16 | 51.58 | 51.78 | -3.98 | < 0.001 | 55.82 | | 51.77 | 50.97 | 49.31 | -6.51 | < 0.001 | 0.152 |
|  | SE | 0.92 | 0.86 | 0.85 | 0.85 | 0.88 |  | 0.93 | | 0.87 | 0.85 | 0.82 | 0.88 |  |  |
| PROMIS Sleep-Related Impairment (T-score) | Mean | 56.40 | 51.63 | 52.99 | 52.95 | -3.45 | 0.001 | 57.65 | | 52.97 | 51.20 | 50.84 | -6.80 | < 0.001 | 0.016 |
|  | SE | 1.20 | 1.10 | 1.12 | 1.12 | 1.03 |  | 1.24 | | 1.15 | 1.10 | 1.10 | 1.04 |  |  |
| RSQ | Mean | 43.74 | 55.71 | 56.37 | 56.26 | 12.52 | < 0.001 | 44.73 | | 58.31 | 59.51 | 62.50 | 17.77 | < 0.001 | 0.611 |
|  | SE | 2.14 | 2.72 | 2.76 | 2.75 | 2.30 |  | 2.22 | | 2.91 | 2.95 | 3.10 | 2.55 |  |  |
| WHO-5 | Mean | 12.52 | 14.90 | 14.55 | 14.79 | 2.27 | < 0.001 | 12.84 | | 15.67 | 15.78 | 16.66 | 3.82 | < 0.001 | 0.258 |
|  | SE | 0.50 | 0.59 | 0.58 | 0.59 | 0.48 |  | 0.52 | | 0.64 | 0.64 | 0.67 | 0.54 |  |  |

Results (estimated means) are generated from generalised mixed-effects models adjusted for age, sex, BMI, and CTTES positive and negative expectancies score.

^a^P-values are generated from repeated measures generalised mixed-effects models adjusted for age, sex, BMI, and CTTES positive and negative expectancies score (time effects day 0 and day 42).

^b^P-values are generated from repeated measures generalised mixed-effects models for age, sex, BMI, and CTTES positive and negative expectancies score (time x group interaction).

**Supplementary Table 4. Change in Self-Report Questionnaires – selected sample (estimated marginal means) (Per Protocol Set)**

|  | | Placebo | | | | | | Magtein^®^ | | | | | | p-value^b^ |
| --- | --- | --- | --- | --- | --- | --- | --- | --- | --- | --- | --- | --- | --- | --- |
|  |  | Day 0 | Day 14 | Day 28 | Day 42 | Change from baseline | p-value^a^ | Day 0 | Day 14 | Day 28 | Day 42 | Change from baseline | p-value^a^ |  |
| PROMIS Sleep Disturbance (T-score) **(≥ 56.5; ≥ 75^th^ percentile)** | N | 15 | | | | | | 22 | | | | | | 0.009 |
|  | Mean | 58.26 | 54.04 | 54.36 | 54.70 | -3.56 | 0.015 | 59.05 | 53.42 | 51.08 | 50.05 | -9.00 | < 0.001 |  |
|  | SE | 1.47 | 1.37 | 1.38 | 1.38 | 1.44 |  | 1.33 | 1.20 | 1.15 | 1.12 | 1.16 |  |  |
| PROMIS Sleep-Related Impairment (T-score)  **(≥ 56.5; ≥ 75^th^ percentile)** | N | 26 | | | | | | 28 | | | | | | 0.014 |
|  | Mean | 58.68 | 53.52 | 55.19 | 55.42 | -3.26 | 0.015 | 60.40 | 55.73 | 53.08 | 53.13 | -7.27 | < 0.001 |  |
|  | SE | 1.31 | 1.19 | 1.23 | 1.24 | 1.33 |  | 1.31 | 1.23 | 1.15 | 1.15 | 1.28 |  |  |
| RSQ  **(below sample median of 44)** | N | 25 | | | | | | 22 | | | | | | 0.879 |
|  | Mean | 36.73 | 52.17 | 50.69 | 51.93 | 15.20 | < 0.001 | 37.13 | 51.75 | 52.62 | 55.18 | 18.05 | < 0.001 |  |
|  | SE | 2.52 | 3.58 | 3.47 | 3.56 | 2.88 |  | 2.67 | 3.72 | 3.78 | 3.97 | 3.24 |  |  |

Results (estimated means) are generated from generalised mixed-effects models adjusted for age, sex, BMI, and CTTES positive and negative expectancies score.

^a^P-values are generated from repeated measures generalised mixed-effects models adjusted for age, sex, BMI, and CTTES positive and negative expectancies score (time effects day 0 and day 42).

^b^P-values are generated from repeated measures generalised mixed-effects models for age, sex, BMI, and CTTES positive and negative expectancies score (time x group interaction).

**Supplementary Table 5. Change in Oura Ring Measures (estimated marginal means) (Per Protocol Set)**

|  |  | Placebo (n=41) | | | | | | | | | Magtein^®^ (n=40) | | | | | | | | | p-value^b^ |
| --- | --- | --- | --- | --- | --- | --- | --- | --- | --- | --- | --- | --- | --- | --- | --- | --- | --- | --- | --- | --- |
|  |  | Week  0 | Week  1 | Week  2 | Week  3 | Week  4 | Week  5 | Week 6 | Change from baseline | p-value^a^ | Week  0 | Week  1 | Week  2 | Week  3 | Week  4 | Week  5 | Week 6 | Change from baseline | p-value^a^ |  |
| Total duration of sleep period (minutes) | Mean | 478.82 | 478.24 | 481.38 | 487.76 | 475.01 | 482.64 | 475.26 | -3.56 | 0.107 | 482.65 | 479.02 | 460.48 | 479.27 | 471.46 | 478.29 | 463.63 | -19.02 | 0.037 | 0.495 |
|  | SE | 9.55 | 9.54 | 9.60 | 9.73 | 9.48 | 9.63 | 9.48 | 8.64 |  | 9.88 | 9.80 | 9.49 | 9.88 | 9.72 | 9.86 | 9.75 | 9.08 |  |  |
| Total sleep time (minutes) | Mean | 414.43 | 411.77 | 416.82 | 424.16 | 410.63 | 418.67 | 409.97 | -4.46 | 0.532 | 409.50 | 409.93 | 393.37 | 409.35 | 400.40 | 408.82 | 398.15 | -11.34 | 0.126 | 0.457 |
|  | SE | 7.84 | 7.79 | 7.89 | 8.03 | 7.77 | 7.92 | 7.76 | 7.14 |  | 7.95 | 7.96 | 7.69 | 8.00 | 7.83 | 7.99 | 7.94 | 7.41 |  |  |
| Total time awake (minutes) | Mean | 63.78 | 65.99 | 64.23 | 63.32 | 64.09 | 63.58 | 64.95 | 1.17 | 0.754 | 73.01 | 69.29 | 67.16 | 70.37 | 70.77 | 69.06 | 65.69 | -7.33 | 0.088 | 0.801 |
|  | SE | 4.40 | 4.55 | 4.43 | 4.36 | 4.42 | 4.38 | 4.48 | 3.74 |  | 5.16 | 4.90 | 4.78 | 5.01 | 5.03 | 4.91 | 4.75 | 4.28 |  |  |
| Light sleep (minutes) | Mean | 248.12 | 244.92 | 251.73 | 254.10 | 246.40 | 249.01 | 244.72 | -3.41 | 0.534 | 247.67 | 250.18 | 235.78 | 254.08 | 244.05 | 249.82 | 238.01 | -9.66 | 0.091 | 0.168 |
|  | SE | 6.26 | 6.18 | 6.35 | 6.41 | 6.22 | 6.28 | 6.18 | 5.47 |  | 6.41 | 6.48 | 6.15 | 6.62 | 6.36 | 6.51 | 6.32 | 5.71 |  |  |
| REM sleep (minutes) | Mean | 89.31 | 89.81 | 88.98 | 92.09 | 90.84 | 92.06 | 89.13 | -0.18 | 0.947 | 87.45 | 88.29 | 85.64 | 83.22 | 85.44 | 86.96 | 87.29 | -0.16 | 0.952 | 0.410 |
|  | SE | 3.38 | 3.40 | 3.36 | 3.48 | 3.44 | 3.48 | 3.37 | 2.63 |  | 3.39 | 3.42 | 3.34 | 3.25 | 3.33 | 3.39 | 3.46 | 2.73 |  |  |
| Deep sleep (minutes) | Mean | 76.59 | 76.42 | 76.03 | 77.55 | 72.94 | 77.30 | 75.29 | -1.30 | 0.580 | 74.19 | 71.29 | 71.74 | 71.93 | 70.55 | 71.30 | 72.62 | -1.57 | 0.515 | 0.849 |
|  | SE | 2.74 | 2.73 | 2.72 | 2.77 | 2.61 | 2.76 | 2.69 | 2.35 |  | 2.72 | 2.61 | 2.65 | 2.65 | 2.60 | 2.63 | 2.73 | 2.40 |  |  |
| Sleep onset latency (minutes) | Mean | 18.02 | 18.08 | 18.07 | 17.29 | 19.69 | 17.18 | 22.33 | 4.30 | 0.033 | 19.13 | 17.80 | 18.44 | 16.08 | 18.44 | 18.00 | 18.74 | -0.39 | 0.842 | 0.681 |
|  | SE | 1.66 | 1.67 | 1.67 | 1.59 | 1.82 | 1.59 | 2.06 | 2.01 |  | 1.81 | 1.68 | 1.76 | 1.54 | 1.76 | 1.72 | 1.84 | 1.97 |  |  |
| Sleep efficiency (%) | Mean | 86.75 | 86.15 | 86.97 | 87.07 | 86.68 | 87.00 | 86.62 | 0.30 | 0.839 | 84.95 | 85.53 | 85.68 | 85.96 | 85.28 | 85.77 | 86.10 | 1.15 | 0.094 | 0.844 |
|  | SE | 0.78 | 0.77 | 0.78 | 0.78 | 0.78 | 0.78 | 0.78 | 0.63 |  | 0.78 | 0.79 | 0.80 | 0.80 | 0.79 | 0.80 | 0.81 | 0.68 |  |  |
| Wake up count (n) | Mean | 5.40 | 5.66 | 5.13 | 5.53 | 5.59 | 5.40 | 5.30 | -0.20 | 0.687 | 5.53 | 5.40 | 5.10 | 5.49 | 5.66 | 5.37 | 5.24 | -0.28 | 0.280 | 0.969 |
|  | SE | 0.28 | 0.30 | 0.27 | 0.29 | 0.29 | 0.28 | 0.28 | 0.24 |  | 0.30 | 0.29 | 0.28 | 0.30 | 0.31 | 0.29 | 0.29 | 0.26 |  |  |
| Average heart rate (bpm) | Mean | 64.12 | 64.31 | 64.04 | 64.34 | 63.74 | 63.92 | 64.45 | 0.29 | 0.547 | 63.16 | 62.68 | 61.76 | 63.31 | 63.60 | 62.83 | 61.46 | -1.70 | 0.003 | 0.008 |
|  | SE | 1.19 | 1.20 | 1.19 | 1.20 | 1.19 | 1.19 | 1.20 | 0.50 |  | 1.21 | 1.20 | 1.18 | 1.21 | 1.22 | 1.20 | 1.18 | 0.56 |  |  |
| Heart rate variability -RMSSD (ms) | Mean | 37.51 | 37.87 | 38.39 | 37.93 | 39.23 | 39.21 | 36.76 | -0.75 | 0.554 | 44.31 | 45.94 | 48.17 | 45.32 | 43.62 | 47.01 | 46.80 | 2.49 | 0.065 | 0.046 |
|  | SE | 3.09 | 3.09 | 3.09 | 3.09 | 3.09 | 3.09 | 3.09 | 1.27 |  | 3.17 | 3.17 | 3.17 | 3.17 | 3.17 | 3.17 | 3.19 | 1.34 |  |  |

Results (estimated means) are generated from generalised mixed-effects models adjusted for age, sex, and BMI.

^a^P-values are generated from repeated measures generalised mixed-effects models adjusted for age, sex, and BMI (time effects week 0 and week 6).

^b^P-values are generated from repeated measures generalised mixed-effects models for age, sex, and BMI (time x group interaction).

**Supplementary Table 6. Correlation between Baseline Oura Ring Scores and Study Outcomes at Baseline (Full Analysis Set)**

|  | | BMI | Systolic BP | Diastolic BP | Age | Raven’s 2 | NIH Total Cognition Composite | Aim Trainer | RSQ | WHO-5 | PROMIS Sleep Disturbance | PROMIS Sleep-Related Impairment |
| --- | --- | --- | --- | --- | --- | --- | --- | --- | --- | --- | --- | --- |
|  | N | 98 | 95 | 95 | 98 | 98 | 98 | 98 | 98 | 98 | 98 | 98 |
| Oura Sleep Score | Pearson Correlation | **-0.259^*^** | -0.101 | -0.009 | -0.169 | 0.056 | -0.037 | 0.099 | 0.037 | 0.036 | 0.079 | 0.093 |
|  | Sig. (2-tailed) | **0.010** | 0.330 | 0.934 | 0.096 | 0.584 | 0.720 | 0.333 | 0.714 | 0.724 | 0.440 | 0.363 |
| Oura Sleep Duration | Pearson Correlation | -0.056 | -0.040 | 0.006 | -0.071 | 0.119 | **0.281^**^** | 0.053 | -0.073 | -0.016 | **0.247^*^** | -0.046 |
|  | Sig. (2-tailed) | 0.587 | 0.703 | 0.956 | 0.488 | 0.242 | **0.005** | 0.606 | 0.475 | 0.878 | **0.014** | 0.654 |
| Oura Total Sleep Duration | Pearson Correlation | -0.154 | -0.067 | -0.007 | **-0.214^*^** | 0.104 | 0.176 | 0.106 | -0.057 | -0.006 | 0.189 | 0.013 |
|  | Sig. (2-tailed) | 0.131 | 0.519 | 0.948 | **0.035** | 0.309 | 0.084 | 0.298 | 0.580 | 0.952 | 0.063 | 0.899 |
| Oura Awake Time | Pearson Correlation | 0.152 | 0.030 | 0.025 | **0.226^*^** | 0.082 | 0.317^**^ | -0.073 | -0.063 | -0.024 | **0.216^*^** | -0.127 |
|  | Sig. (2-tailed) | 0.136 | 0.771 | 0.813 | **0.025** | 0.424 | 0.001 | 0.477 | 0.539 | 0.813 | **0.033** | 0.212 |
| Oura Light Sleep | Pearson Correlation | 0.041 | 0.000 | 0.013 | -0.077 | 0.032 | **0.230^*^** | 0.054 | -0.013 | -0.026 | 0.113 | -0.032 |
|  | Sig. (2-tailed) | 0.691 | 1.000 | 0.898 | 0.453 | 0.752 | **0.023** | 0.599 | 0.898 | 0.798 | 0.268 | 0.753 |
| Oura REM Sleep | Pearson Correlation | **-0.298^**^** | -0.172 | -0.019 | -0.178 | 0.085 | 0.037 | 0.050 | -0.082 | 0.046 | 0.196 | -0.009 |
|  | Sig. (2-tailed) | **0.003** | 0.095 | 0.856 | 0.080 | 0.404 | 0.717 | 0.625 | 0.424 | 0.654 | 0.053 | 0.927 |
| Oura Deep Sleep | Pearson Correlation | **-0.254^*^** | 0.005 | -0.033 | **-0.318^**^** | 0.170 | -0.051 | 0.168 | -0.055 | -0.014 | 0.100 | 0.151 |
|  | Sig. (2-tailed) | **0.012** | 0.964 | 0.748 | **0.001** | 0.094 | 0.621 | 0.098 | 0.592 | 0.888 | 0.326 | 0.137 |
| Oura Sleep-Onset Latency | Pearson Correlation | 0.118 | 0.053 | 0.055 | **0.281^**^** | 0.071 | **0.305^**^** | -0.019 | -0.051 | -0.039 | 0.069 | -0.158 |
|  | Sig. (2-tailed) | 0.247 | 0.610 | 0.596 | **0.005** | 0.487 | **0.002** | 0.855 | 0.619 | 0.704 | 0.499 | 0.120 |
| Oura Sleep Efficiency | Pearson Correlation | **-0.225^*^** | -0.072 | -0.001 | **-0.267^**^** | 0.005 | **-0.250^*^** | 0.116 | 0.010 | -0.021 | -0.161 | 0.112 |
|  | Sig. (2-tailed) | **0.026** | 0.487 | 0.992 | 0.008 | 0.959 | **0.013** | 0.254 | 0.920 | 0.84 | 0.112 | 0.271 |
| Oura Wake Up Count | Pearson Correlation | 0.015 | **0.237^*^** | 0.171 | -0.176 | -0.094 | 0.173 | 0.044 | 0.110 | 0.071 | 0.015 | **-0.235^*^** |
|  | Sig. (2-tailed) | 0.881 | **0.021** | 0.098 | 0.084 | 0.358 | 0.088 | 0.666 | 0.283 | 0.485 | 0.887 | **0.020** |
| Oura Average Heart Rate During Sleep | Pearson Correlation | 0.173 | 0.093 | **0.388^**^** | -0.068 | -0.043 | 0.034 | -0.120 | **-0.341^**^** | **-0.228^*^** | **0.229^*^** | **0.281^**^** |
|  | Sig. (2-tailed) | 0.088 | 0.371 | **0.000** | 0.503 | 0.677 | 0.739 | 0.237 | **0.001** | **0.024** | **0.023** | **0.005** |
| Oura Average RMSSD During Sleep | Pearson Correlation | -0.188 | -0.086 | **-0.284^**^** | **-0.273^**^** | 0.090 | 0.049 | **0.257^*^** | 0.007 | 0.055 | -0.024 | -0.063 |
|  | Sig. (2-tailed) | 0.063 | 0.408 | **0.005** | **0.007** | 0.377 | 0.630 | **0.011** | 0.945 | 0.593 | 0.816 | 0.535 |
